# Three-dimensional structure of human cyclooxygenase (*h*COX)-1

**DOI:** 10.1038/s41598-021-83438-z

**Published:** 2021-02-22

**Authors:** Morena Miciaccia, Benny Danilo Belviso, Mariaclara Iaselli, Gino Cingolani, Savina Ferorelli, Marianna Cappellari, Paola Loguercio Polosa, Maria Grazia Perrone, Rocco Caliandro, Antonio Scilimati

**Affiliations:** 1 Department of Pharmacy - Pharmaceutical Sciences, University of Bari “Aldo Moro”, Via E. Orabona 4, 70125 Bari, Italy; 2 Istituto di Cristallografia, Consiglio Nazionale delle Ricerche, Via Amendola 122/o, 70126 Bari, Italy; 3 Department of Biochemistry and Molecular Biology, Thomas Jefferson University, 1020 Locust Street, Philadelphia, PA 19107 USA; 4 Department of Biosciences, Biotechnologies, and Biopharmaceutics, University of Bari “Aldo Moro”, Via E. Orabona 4, 70125 Bari, Italy

**Keywords:** Enzymes, Membrane proteins, Isolation, separation and purification, Chromatography, Protein purification, Structural biology, X-ray crystallography

## Abstract

The beneficial effects of Cyclooxygenases (COX) inhibitors on human health have been known for thousands of years. Nevertheless, COXs, particularly COX-1, have been linked to a plethora of human diseases such as cancer, heart failure, neurological and neurodegenerative diseases only recently. COXs catalyze the first step in the biosynthesis of prostaglandins (PGs) and are among the most important mediators of inflammation. All published structural work on COX-1 deals with the ovine isoenzyme, which is easier to produce in milligram-quantities than the human enzyme and crystallizes readily. Here, we report the long-sought structure of the human cyclooxygenase-1 (*h*COX-1) that we refined to an R/R_free_ of 20.82/26.37, at 3.36 Å resolution. *h*COX-1 structure provides a detailed picture of the enzyme active site and the residues crucial for inhibitor/substrate binding and catalytic activity. We compared *h*COX-1 crystal structure with the ovine COX-1 and human COX-2 structures by using metrics based on Cartesian coordinates, backbone dihedral angles, and solvent accessibility coupled with multivariate methods. Differences and similarities among structures are discussed, with emphasis on the motifs responsible for the diversification of the various enzymes (primary structure, stability, catalytic activity, and specificity). The structure of *h*COX-1 represents an essential step towards the development of new and more selective COX-1 inhibitors of enhanced therapeutic potential.

## Introduction

Prostaglandin endoperoxide H synthases (PGHSs), also known as cyclooxygenases (COXs), catalyze the conversion of free arachidonic acid (AA), released from cell membrane upon mitogen or mechanical stimuli, into Prostaglandin H_2_ (PGH_2_), that represents the committed step in the biosynthesis of prostaglandins, prostacyclin, and thromboxane^1^. The known COX isoforms (COX-1 and COX-2) are the product of two different genes^2,3^. The gene that encodes for COX-1 is considered a “housekeeping gene” due to its low levels of expression in most cell types. However, high levels of constitutive COX-1 expression have been detected in the gastric mucosa and platelets. In contrast to COX-1, COX-2 expression is typically inducible in response to pro-inflammatory events and growth factors^2^. COX-1 and COX-2 are of particular interest because they are the major target of non-steroidal anti-inflammatory drugs (NSAIDs)^3^, which bind to the COX active site preventing that AA reaches the catalytic pocket^1^ and, hence, prostaglandins biosynthesis. Prostaglandins, in particular PGE_2_, are crucial mediators implicated in inflammation, angiogenesis and support the growth of several solid tumors^4,5^. Therefore, inhibition of COXs is relevant to reduce inflammation, pain, and fever, as well as cancer progression.

Compared to COX-2, COX-1 involvement in tumorigenesis has been less studied^5^. Recent work demonstrates a direct involvement of COX-1 in breast, ovarian^6^, head and neck cancer, renal cell carcinoma, and hematological tumors^5,7,8^. For all these cancers, overexpression of COX-1 could be exploited as a diagnostic biomarker^9–12^.

X-ray crystallographic analysis has proven a powerful tool to investigate COX active site and in unraveling the binding mode of COX inhibitors. The ovine isoform of COX-1 (*o*COX-1) has been crystallographically studied over a dozen times in complex with inhibitors and substrates^13^. More difficult has been working with the human isoform (*h*COX-1). Herein, we describe the expression and purification of *h*COX-1 from baculovirus-infected insect cells that yielded milligram-quantities of the enzyme. Ectopically expressed *h*COX-1 yielded crystals suitable for structure determination by using X-ray crystallographic analysis.

## Results

### *h*COX-1 expression and purification

The production of milligram quantities of correctly folded and active *h*COX-1 is vital for the structural, biochemical and pharmacological analysis of human COX-1, a key target of a plethora of pharmaceuticals, clinically used to treat several human diseases. In this study, we used the BacPAK™ Baculovirus Expression System to express large quantities of folded and post-translationally modified *h*COX-1 suitable for structural analysis.

*h*COX-1 is 599 amino acids long and includes at the *N*-terminus a 24-amino acid signal peptide that targets the protein to the endoplasmic reticulum for post-translational modification. We aimed to construct a recombinant *h*COX-1 baculovirus that expresses the protein suitable for nickel affinity chromatography purification by using the BacPAK Baculovirus Expression System. The *h*COX-1 DNA was PCR amplified from a previously generated pFastBac-*h*COX-1 plasmid that also contains an 8xHistidine tag (8XHis-tag) placed just after the signal peptide, followed by a 7-amino acid TEV protease cleavage site. The amplification was carried out using the proofreading Phusion HF DNA polymerase and specific primers containing at their 5′ ends BglII and NotI restriction sites. The amplified DNA was evaluated by agarose gel electrophoresis, showing, as expected, a single product of about 1800 bp (Supplementary Fig. 1). The purified products were digested by BglII and NotI restriction enzymes and ligated with the shuttle plasmid pBacPAK9. Then, the ligation products were amplified in bacteria and, once extracted, they were restriction digested to assess the presence of the target gene; the correct sequence of the insert was verified by sequencing of both strands. The cloning efficiency was about 100% (Supplementary Fig. 2). To generate the recombinant expression virus, the transfer vector pBacPAK9 containing 8XHis-tag *h*COX-1 cDNA was transfected into *Spodoptera frugiperda* cells along with Bsu36 I-digested BacPAK6 Viral DNA; an in vivo homologous recombination between the plasmid DNA and viral DNA occurred, producing recombinant baculovirus^14^.

To isolate a pure clone of a recombinant virus, the supernatants from co-transfection containing progeny viruses were collected and subjected to plaque assay to produce individual plaques (plaque-pick). All plaque-picks were first amplified to produce Virus Stock I (VSI), then viral DNA was extracted and analyzed by PCR, confirming that all clones were positive^15^ (Supplementary Fig. 3).

Hereafter, VSI was amplified twice, according to the manufacturer’s instructions, to maximize the yield of recombinant protein production. A scale-up production and purification protocol for the *h*COX-1 isoenzyme was substantially the same as previously described for *o*COX-1^16^.

SDS-PAGE analysis of metal affinity chromatography fractions revealed *h*COX-1 eluted around 0.25 M imidazole; the eluted protein was highly pure as no low molecular weight species were detectable after Blue Coomassie staining of the gel (Supplementary Fig. 4).

*h*COX-1 protein fractions were pooled and further purified by performing in-solution cleavage, with direct addition of TEV_6His_ protease to the eluted fraction. Following cleavage, the protein solution was loaded on nickel beads, where his-tag and TEV_6His_ were recaptured on the resin. Untagged *h*COX-1 was collected in the flow-through, buffer exchanged and concentrated to 10.4 mg ml^−1^.

### Stability, catalytic activity, and specificity of *o*COX-1 and *h*COX-1

**Figure 1:**
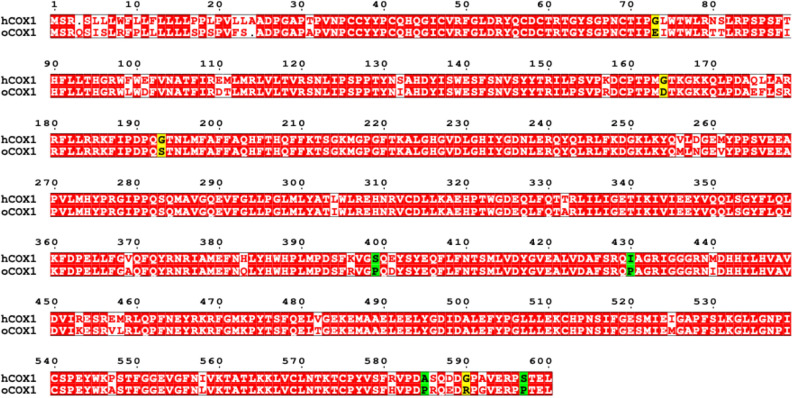
Alignment of human and ovine COX-1. Amino acid sequences of COX-1 from human, *h*, (NM_000962.4) and ovine, *o*, (NM_001009476.1) were aligned with the ClustalW program. The alignment was performed at NPS@ Web server of the PBIL using GONNET weight matrix and formatted with ESPript 3.0^21^. The red boxes include identical amino acids in both sequences. Unconserved residues are included in the white boxes. Among these amino acids, the proline residues present only in *o*COX-1 are shown in the green boxes, while the glycine residues present only in *h*COX-1 are displayed in the yellow boxes.

The availability of both ovine and human COX-1 let us to verify if their stability, catalytic activity, and specificity are similar or different. In particular, their thermal and chemical stability was determined at + 4, − 20, and − 80 °C. Within three months, both enzymes stored at the first two temperatures exhibited an impaired catalytic activity, that in turn remains unchanged at − 80 °C for the same period of time. Their specific activity, instead, was determined in the presence of arachidonic acid and one of the different well-known NSAIDs-COX-1 inhibitors (Table 1), such as mofezolac (a diarylisoxazole sold in Japan as Disopain® to treat rheumatoid arthritis and that could be worldwide repurposed for neurodegenerative diseases with a marked neuroinflammatory component)^17,18^; the ibuprofen, one of the arylpropanoic acids (profen-series) representative of the most commonly prescribed NSAIDs, and indomethacin belonging to the chemical class of indole-acetic acids actually used for treating mild to moderate acute pain in adult. The specific activities found were a little higher for *h*COX-1 than *o*COX-1, even if the two values are very similar in all three cases. The similarity could be ascribed to the high sequence identity (92%) among the two proteins (Fig. 1).

**Table 1:** *o*COX-1 IC_50_ and *h*COX-1 IC_50_, and the specific activity of the two enzymes.

Substrate	Inhibitor	IC_50_ (µM)^a^		EPI^b^	Specific activity (μmol min^−1^ mg^−1^)	
		*o*COX-1	*h*COX-1		*o*COX-1	*h*COX-1
Arachidonic acid (100 µM)		9.2	23	0.4	3.5	4.5
		31	44	0.7	5.9	8.2
		158	2.9	54	3.9	4.4

^a^Determined by Cyclooxygenase O_2_ Electrode Assay.

^b^EPI (enzyme preference index) = *o*COX-1 IC_50_/*h*COX-1 IC_50_.

Mofezolac inhibited *o*COX-1 to a greater extent than the corresponding human enzyme (*o*COX-1 IC_50_ = 9.2 µM; *h*COX-1 IC_50_ = 23 µM), ibuprofen *o*COX-1 IC_50_ and *h*COX-1 IC_50_ are comparable being 31 and 44 µM, respectively. The indomethacin inhibitory potency towards *h*COX-1 was found to be of two orders of magnitude higher than the ovine enzyme (*o*COX-1 IC_50_ a = 158 µM and *h*COX-1 IC_50_ = 2.9 µM) and with an enzyme preference index (EPI) for *h*COX-1 equal to 54 (Table 1).

### *h*COX-1 crystallization experiments

*h*COX-1 crystallization experiments were carried out by following the crystallization procedure reported for *o*COX-1^19^. The best diffracting crystals (Supplementary Fig. 5) were obtained in the presence of 0.5–0.6 M lithium chloride, 10-11 mg/ml of protein concentration, 0.7 M sodium citrate, and 1:1 molar ratio between protein and heme (Fe^3+^–protoporphyrin IX).

### *h*COX-1 crystal structure

**Figure 2:**
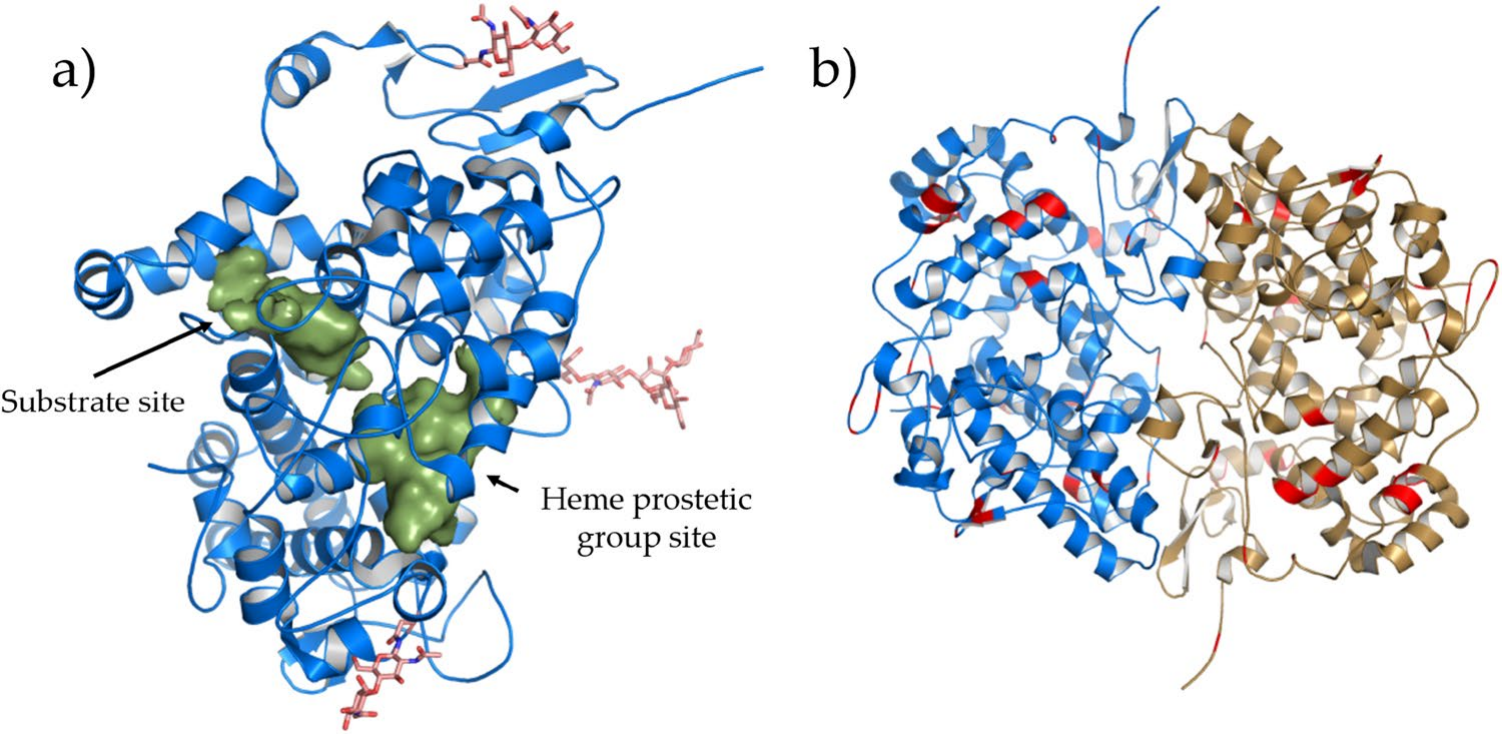
*h*COX-1 crystal structure (PDB code 6Y3C). (**a**) Asymmetric unit of *h*COX-1: protein is shown in blue cartoon, and glycosylation along with glycosylation-linked asparagine residues are in pink stick representation. The solvent excluded surface (determined at solvent radius 1.4 Å) of heme and substrate site is shown in green. (**b**) Biological unit of *h*COX-1: the two protein molecules forming a functional dimer are shown in cartoon representation in blue and sand color. Different residues between *h*COX-1 and *o*COX-1 are in red color.

*h*COX-1 crystals were subjected to X-ray diffraction experiments. Data collection and refinement statistics are shown in Table 2. The data resolution cutoff of 3.36 Å allowed meeting requirements of < I/σ(I) >  > 1.5 and CC_1/2_ > 50% for the high-resolution shell. The data resolution limit and the high value of R_sym_ in the high-resolution shell could not be improved by varying crystallization conditions and trying different crystals. The *h*COX-1 crystal shows P 6_5_22 symmetry, and its asymmetric unit contains a single protein molecule (Fig. 2a). According to the PISA server^20^, the protein in the asymmetric unit interacts with a symmetry-related molecule (symmetry operation y, x, − z-1/3) to form a dimeric biological unit (Fig. 2b), whose interaction interface is about 26% of the solvent-exposed area of the monomer. The high free energy calculated for dimer dissociation (17.6 kcal/M) suggests that such a biological unit is thermodynamically stable. Diffraction data quality is enough to model the expected *N*-acetyl glucosamine (NAG) chain on N68, N410, N144 that are shown in stick representation in Fig. 2a. The validation procedure of the Protein Data Bank server shows quite a constant electron density coverage along the entire protein chain. Poorly covered residues having RSRZ > 2, where RSRZ is the difference between the experimental electron density and that obtained by the model, normalized by residue type and resolution, are only 4% of the all protein residues and are located at the *N*-terminus and in the regions that span 130–150 and 200–213. Such a density coverage points out the good agreement between data and the modeled protein chain. The final model has good geometry relatively to the resolution, with 18% of residues having one geometry outliers, and only 1% showing two outliers.

**Table 2:** Data collection and refinement statistics of the *h*COX-1 crystal structure (PDB code 6Y3C). Outer shell statistics are in parenthesis. For the sake of completeness, statistics before and after anisotropy correction by the STARANISO server are shown.

**Data collection**		
Wavelength (Å)	0.9795	
Space group	P 6_5_22	
Cell constants a, c (Å)	182.11 103.24	
	Before STARANISO	After STARANISO
Resolution range (Å)	91.05–3.36 (3.45–3.36)	91.05–3.36 (3.48–3.36)
Completeness (%)	99.9 (99.9)	81.1 (25.16)
Redundancy	39.1 (42.2)	39.1 (42.2)
Total number of reflections	582,091 (45,649)	581,081 (61,045)
Unique reflections	14,886 (1081)	14,838 (363)
R sym (%)	0.467 (4.244)	0.493 (3.356)
R meas (%)	0.473 (4.295)	0.499 (3.578)
< I/σ(I) >	14.3 (1.4)	14.3 (1.5)
CC_1/2_ (%)	99.9 (63.6)	99.9 (69.0)
Wilson B-factor (Å^2^)	94.32	94.47
**Refinement**		
R/Rfree (%)	20.82/26.37 (29.17 /36.30)	
MolProbity score	1.59	
Clashscore	7	
Average B-factor (Å^2^) (protein atoms)	100.54	
Average B-factor (Å^2^) (water atoms)	61.12	
Average B-factor (Å^2^) (heterogen atoms)	111.55	
Protein atoms	4518	
Water molecules	17	
Heterogen atoms	155	
RMSD bonds length (Å)	0.002	
RMSD bond angles (°)	0.53	
Ramachandran favored (%)	96.76	
Ramachandran allowed (%)	3.24	
Ramachandran outlier (%)	0.00

**Figure 3:**
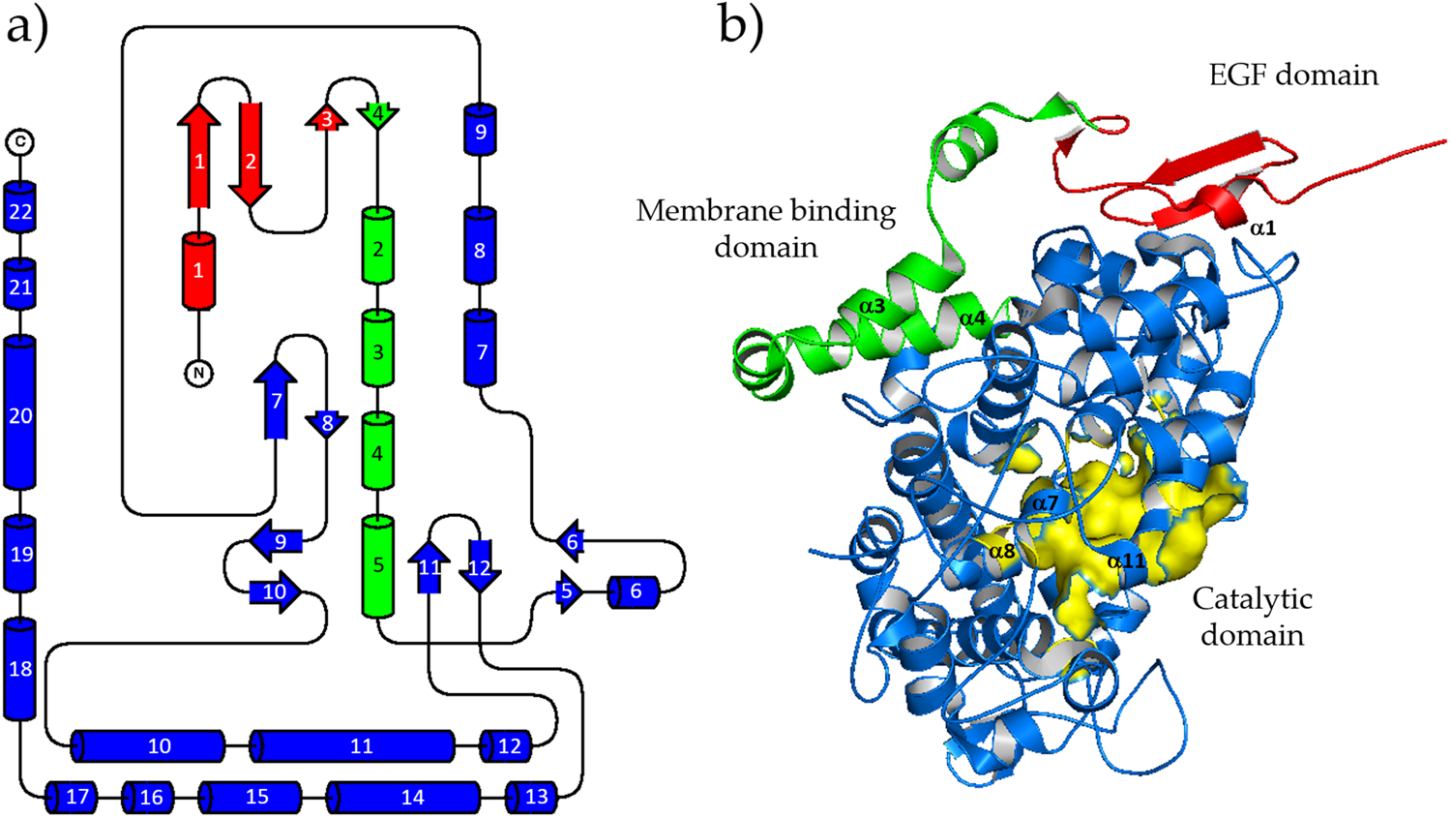
*h*COX-1 folding. (**a**) *h*COX-1 topology diagram: secondary structure elements are shown in red, green, and blue for EGF, membrane-binding domain, and catalytic domain, respectively. β-strands and α-helixes are shown as arrows and cylinders, respectively; (**b**) *h*COX-1 domains are shown in cartoon representation in the same color code of (**a**). Solvent excluded surface of the catalytic domain is in yellow color.

Secondary structure elements and their mutual orientation are shown in Fig. 3a. *h*COX-1 protein fold reveals the peculiar arrangement of an epidermal growth factor (EGF) and a membrane-binding domain placed on the surface of a catalytic domain, quite far from the heme site Fig. 3b. With respect to the EGF domain, the membrane-binding domain has more interactions with the catalytic domain and, particularly, with the region forming the substrate pocket of the protein. Such protein folding follows that of the *o*COX-1, the protein having the highest sequence identity with *h*COX-1, and that of the *h*COX-2.

**Figure 4:**
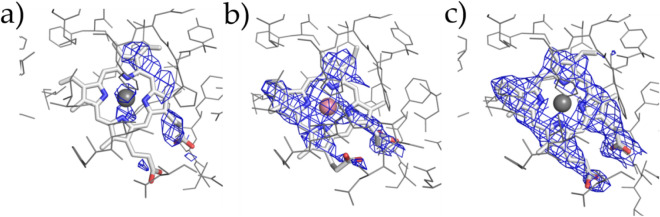
Polder map of heme molecule. Protein residues are shown in gray lines and heme molecules are as gray sticks. (**a**–**c**) show the polder map contoured at 2.5σ above background for *h*COX-1 (PDB code 6Y3C), *o*COX-1 (PDB code 1U67), and *o*COX-1 (PDB code 5U6X).

The heme molecule, which is important for COX activity, was added at 1:1 molar ratio with *h*COX-1 monomer before crystallization. Such a stoichiometric quantity of heme promoted crystallization resulted in poor electron density in the region where heme molecule is expected. Refining the structure with the heme molecule resulted in an increase of R/R_free_ from 20.82/26.37 to 21.50/26.58. In Fig. 4, the polder map calculated by omitting the heme group in our structure is compared with those calculated in the same way for two previously determined *o*COX-1 structures [1U67 (P 6_5_22 symmetry) and 5U6X (P 6_5_ symmetry)]. The density for heme is much weaker in the *h*COX-1 than the other two structures, confirming the poor occupancy of this prosthetic group in our structure. Interestingly, the polder electron density covers only partially the heme molecule, mainly, the portion that interacts with protein residues. Such a feature is compatible with a flexible molecule rather than a stable molecule at low crystallographic occupancy. To make sure that such electron density features related to the heme molecule are not an artifact due to an incorrect choice of space group, data were reprocessed in the alternative space group P 6_5_, suggested by the indexing process, where the two protein chains can have different conformations, but no difference was observed. Unable to confirm the presence of the heme in our crystal structure, we decided not to include such a prosthetic group in the final model.

**Figure 5:**
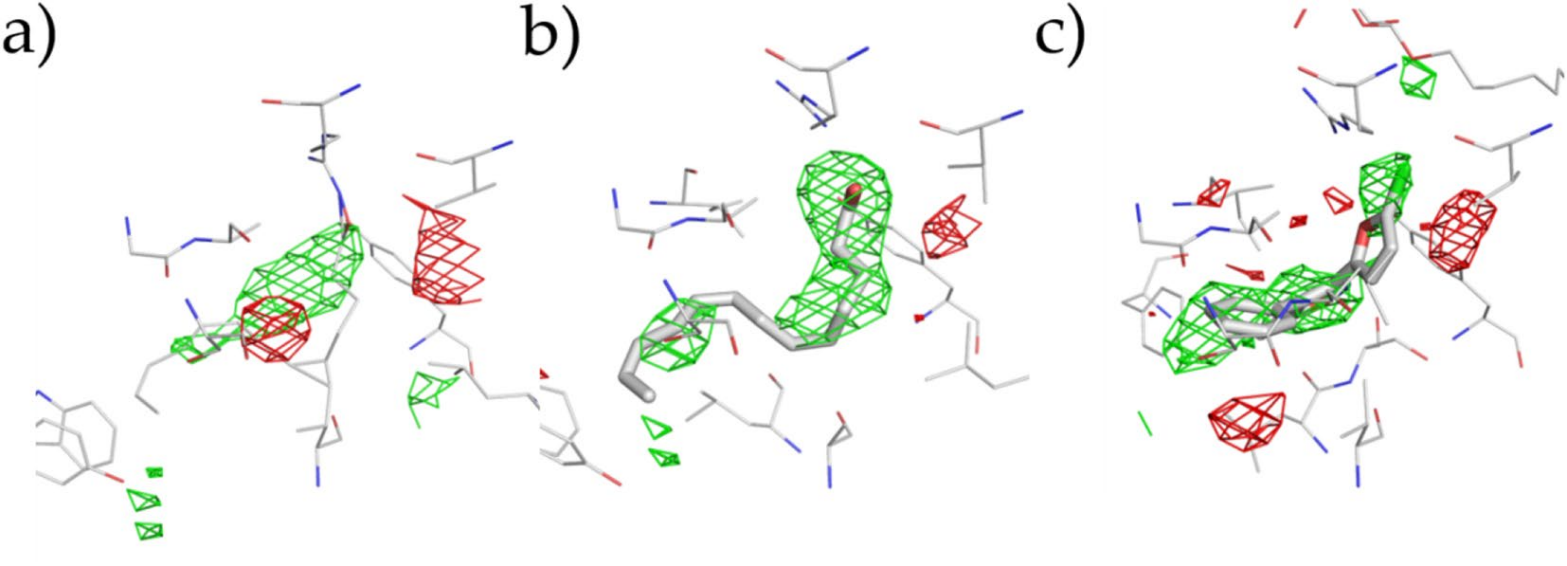
Fourier difference (Fo-Fc) electron density map contoured at >|3σ| (positive and negative peaks are in green and red mesh, respectively) in the substrate site for (**a**), 6Y3C (*h*COX-1), (**b**) 1U67 (*o*COX-1:AA complex crystal), and (**c**) 5U6X (*o*COX-1:P6 complex crystal). Enzymatic protein residues are in gray lines and AA or P6 in the substrate site in gray sticks. Fourier difference electron density map was obtained by removing ligand molecules from the structures.

Another region of the enzyme that deserves attention is the substrate site. Even though our *h*COX-1 crystals were obtained in the absence of molecules that bind the substrate site, a peak of difference (Fo-Fc) electron density can be observed in this region (Fig. 5a). Fo-Fc density map was compared with that obtained by removing arachidonic acid (COXs endogenous substrate) and P6 (highly selective COX-1 inhibitor)^22–24^ ligand from 1U67 (Fig. 5b)^13^ and 5Y6X (Fig. 5c)^13^, respectively. Although less defined, the peak in our structure shows similar shape and orientation to those in the other two structures. All attempts to fit and refine arachidonic acid, or molecules present in the crystallization cocktail, were unsuccessful. This prompted us not to model this electron density peak in the final structural model.

### Comparative analysis of *h*COX-1, *o*COX-1 and *h*COX-2 crystal structures

There are twenty-nine crystal structures of *o*COX-1 deposited in the Protein Data Bank (thirteen of which crystallized in the space group I 222, nine in the space group P 6_5_, and seven in P 6_5_22) and seven crystal structures for *h*COX-2 (all crystallized in the space group I 222). Differences in protein conformation among these crystal structures could be due to different crystallization conditions, crystal packing, or protein sequence. While the first two are properties of the crystal state, the latter one is related to the intrinsic conformation of the protein. In the case of COX proteins, deciphering the intrinsic conformation of the human enzyme is particularly important for drug design and development. COX-1 is a functional dimer, and dimerization is strictly required for structural integrity and activity^25^. Nonetheless, it remains unclear if the COX-1 dimer is built by conformationally equivalent or *quasi*-equivalent protomers of COX. With this rationale in mind, we performed a comparative analysis of our *h*COX-1 with the crystal structures of *o*COX-1 and *h*COX-2 deposited in the PDB using different metrics and considering the chains of the biological unit of each structure separately.

As a first step, we compared the experimental coordinate errors of the structural models, as estimated by the diffraction-data precision indicator (DPI) (Supplementary Fig. 6). Our *h*COX-1 crystal structure holds a coordinate error of 0.59 Å, mainly due to the medium data resolution (3.36 Å). Comparable errors are shown by *o*COX-1 crystal structures with P 6_5_22 (average error of 0.41 Å) and I 222 symmetry (average error of 0.46 Å). In this latter case, the spread of DPI values is very large, ranging from 0.10 Å for 2AYL and 1Q4G to 1.24 Å for 1PGF and 1PGG. Crystal structures of *h*COX-2 and *o*COX-1 with P 6_5_ symmetry are systematically more precise, having, in both cases, an average error of 0.28 Å. The systematic trend of experimental errors among COXs reflects the quality of diffraction data, and suggests the following trend: first, *h*COX-1 is the most challenging isoform to crystallize; second, COX-2 is intrinsically more stable than COX-1 and hence more likely to crystallize; third, among *o*COX-1 structures deposited in the database, there is an empirical correlation between model accuracy and crystal symmetry with the COX-1 structure solved in space P 6_5_ being the most accurate, followed by P 6_5_22 and finally I 222.

### Comparison based on Cartesian coordinates

**Figure 6:**
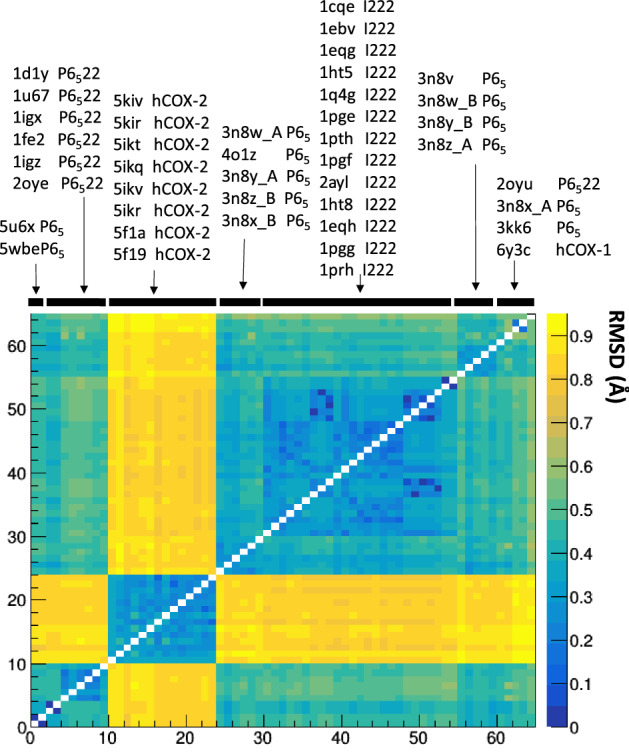
Matrix of the Cα pairwise RMSD values calculated between individual chains of the 37 structures of *h*COX-1, *o*COX-1, and *h*COX-2 present in the PDB. Chains are ordered based on a hierarchical clustering algorithm having RMSD as distance metrics. They are labelled on the top by using the PDB code, the type (*h*COX-1 or *h*COX-2), the space group (only for *o*COX-1), and the chain letter (A or B are reported only if they do not fall in the same cluster).

We superimposed each independent chain of *h*COX-1, *o*COX-1, and *h*COX-2 with each other to generate a matrix (65 chains × 65 chains) of overall Cα RMSD, which was subjected to hierarchic clustering. Despite the large heterogeneity of diffraction data quality of COXs crystals (Supplementary Fig. 6), the crystal structures that are homogeneous by protein, source, and crystallographic symmetry were grouped consistently in the resulting matrix (Fig. 6), as they have similar RMSD values. It can be noted that the two chains of the same crystal structure are grouped in most of the cases, indicating that the functional dimer is preferentially made of two conformationally-equivalent units. Moreover, Fig. 6 clearly shows the cluster of *h*COX-2 chains, which are very different from the *o*COX-1 chains, and the clusters of *o*COX-1 chains with P 6_5_22 and I 222 symmetry, pointing out the high structural similarity (inter-cluster RMSD < 0.4 Å) of these three sets of structures.

*o*COX-1 structures with P 6_5_ symmetry are spread into three clusters, indicating the higher structural heterogeneity related to this crystal packing, where two symmetry-independent chains form the biological unit. A further cluster has slightly higher inter-cluster RMSD values (between 0.4 and 0.6 Å) and includes the following outliers: our *h*COX-1 crystal structure, 2OYU, a *o*COX-1 structure with P 6_5_22 symmetry, the chain A of the structure 3N8X and the structure 3KK6, both *o*COX-1 crystallized in P 6_5_. The papers related to these two latter outliers show that data merging by using P 6_5_ and P 6_5_22 provides comparable R_sym_ values^26,27^, a feature that could explain why these structures group with *o*COX-1 structures crystallized in P 6_5_22 rather than with those crystallized in P 6_5_. Interestingly, the sum of all RMSD values obtained in the case of the human isoform is the highest compared to those obtained for the other COX-1 structures (Supplementary Fig. 7). In the case of pairwise comparison between *o*COX-1 structures and our *h*COX-1, the average of the RMSD values is ~ 0.5 Å, and it is ~ 0.8 Å in the case of comparison with *h*COX-2 crystal structures, despite the low sequence identity (60%).

**Figure 7:**
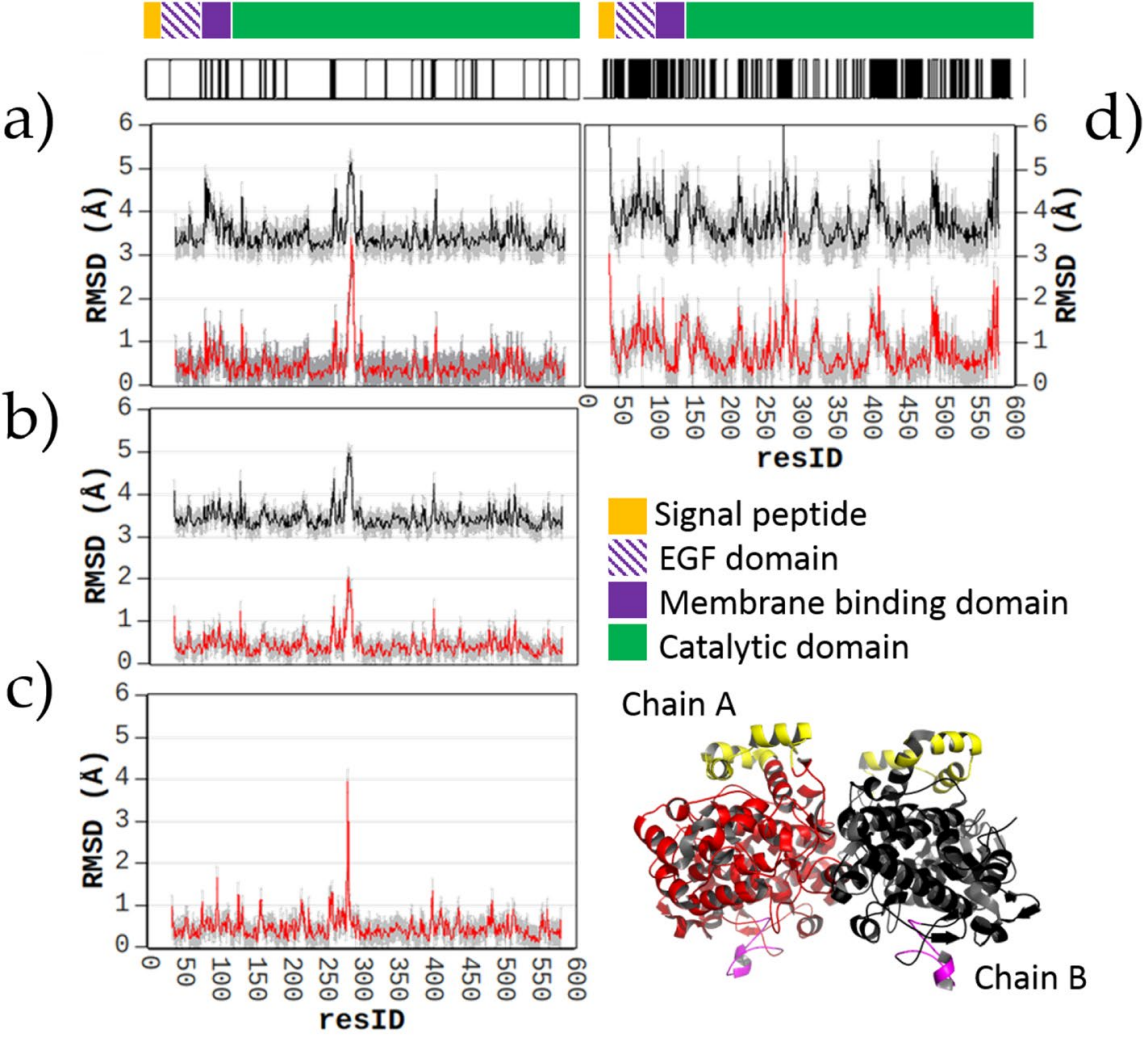
Average residue-by-residue RMSD values calculated after superposition of *h*COX-1 (6Y3C) with (**a**) *o*COX-1 crystallized in the I 222 space group (PDB codes 1Q4G, 2AYL, 1PTH, 1PRH, 1PGG, 1PGE, 1PGF, 1HT8, 1EQH, 1HT5, 1CQE, 1EBV, 1EQG), (**b**) *o*COX-1 crystallized in the P 6_5_ space group (PDB codes 3KK6, 3N8Z, 3N8X, 5WBE, 3N8V, 4O1Z, 3N8Y, 3N8W, 5U6X), (**c**) *o*COX-1 crystallized in the P 6_5_22 space group (PDB codes 1U67, 2OYE, 2OYU, 1IGZ, 1DIY, 1FE2, 1IGX), and (**d**) *h*COX-2 crystallized in the I 222 space group (PDB codes 5F19, 5IKQ, 5IKT, 5KIR, 5F1A, 5IKR, 5IKV, 5KIR) to the *h*COX-1 (6Y3C) crystal structure. Standard deviation for each residue is shown in gray color. RMSD values related to chain A and chain B (nomenclature refers to that adopted for 5U6X shown at bottom right in the figure) are shown in red and black, respectively. Here, regions 70–110 and 270–290 are shown in yellow and magenta color. In the case of *o*COX-1 crystallized in P 6_5_22, the biological unit contains a homo-conformational dimer, therefore only chain A is shown. The numbering of residues refers to *h*COX-1 structure. On the top of the figure, variations between *h*COX-1 and *o*COX-1 (left) and *h*COX-2 (right) are shown, along with *h*COX-1 domains.

An in-depth view of the above structural deviations is provided in Fig. 7, where the *h*COX-1 crystal structure is used as a reference for pairwise superposition, and the residue-by-residue averaged RMSD values for each group of structures are plotted against the residue number. It can be noted that the RMSD values of A (red) and B (black) chains do not show significant differences within the same group. A second observation regards specific regions of the protein affected by high RMSD values. By focusing on the left column (superposition between *h*COX-1 and *o*COX-1 structures), it is possible to observe that the region between 270 and 290 in the catalytic domain is systematically affected by high RMSD values regardless the crystal symmetry. We verified that this very flexible region is mainly responsible for the clustering shown in Fig. 6, as it holds the same conformation in crystal structures within the same cluster, and slightly different conformations for inter-cluster structures (Supplementary Fig. 8).

Interestingly, such a region is poor of residue variations among *h*COX-1 and *o*COX-1; thus, conformational changes are unlikely related to differences in the protein sequence. Differently from the region 270–290, residues between 70 and 110, located in the first two α-helices of the membrane-binding domain, show high RMSD values only compared to *o*COX-1 protein crystallized in the space group I 222. We verified that the same feature arises when comparing *o*COX-1 crystal structures with P 6_5_22 (the same symmetry of our *h*COX-1 crystal structure) or P 6_5_ symmetry with those with I 222 symmetry (Supplementary Fig. 8). Thus, this conformational variation was ascribed to the different effect that crystal packing exerts on the protein conformation in an orthorhombic versus hexagonal lattice. It is worth noting that the same effect does not exist if our *h*COX-1, or *o*COX-1 with P 6_5_22 symmetry if compared with *o*COX-1 crystallized in the P 6_5_ space group. Such a result can be explained by considering that these structures have similar unit cells and crystal packing and differ only in the extension of the asymmetric unit. In addition to crystal packing, also differences in ligand interactions could contribute to the high value of RMSD found in the region 70–110. Indeed, ligands found for *o*COX-1 in I 222 are localized in different way with respect to the citrate ions found in our structure (Supplementary Fig. 9, Supplementary Discussion 1). Similar differences can be observed also by comparing our structure with *h*COX-2. It is worth noting that, since differences in crystal packing and ligand interaction are not directly related to the protein sequence, the high values of RMSD observed in the region 70–110 are unlikely related to intrinsic protein conformation, thus they should not affect inhibitor binding.

When comparing *h*COX-1 and *h*COX-2 (Fig. 7d), it can be noted that in addition to the regions 270–290 and 70–110, highlighted in the comparison between *h*COX-1 and *o*COX-1, also regions 125–175 (*N*-terminus of the catalytic domain), 400–420 and 470–520 both included in the catalytic domain show large deviations.

### Comparison based on backbone dihedral angles

**Figure 8:**
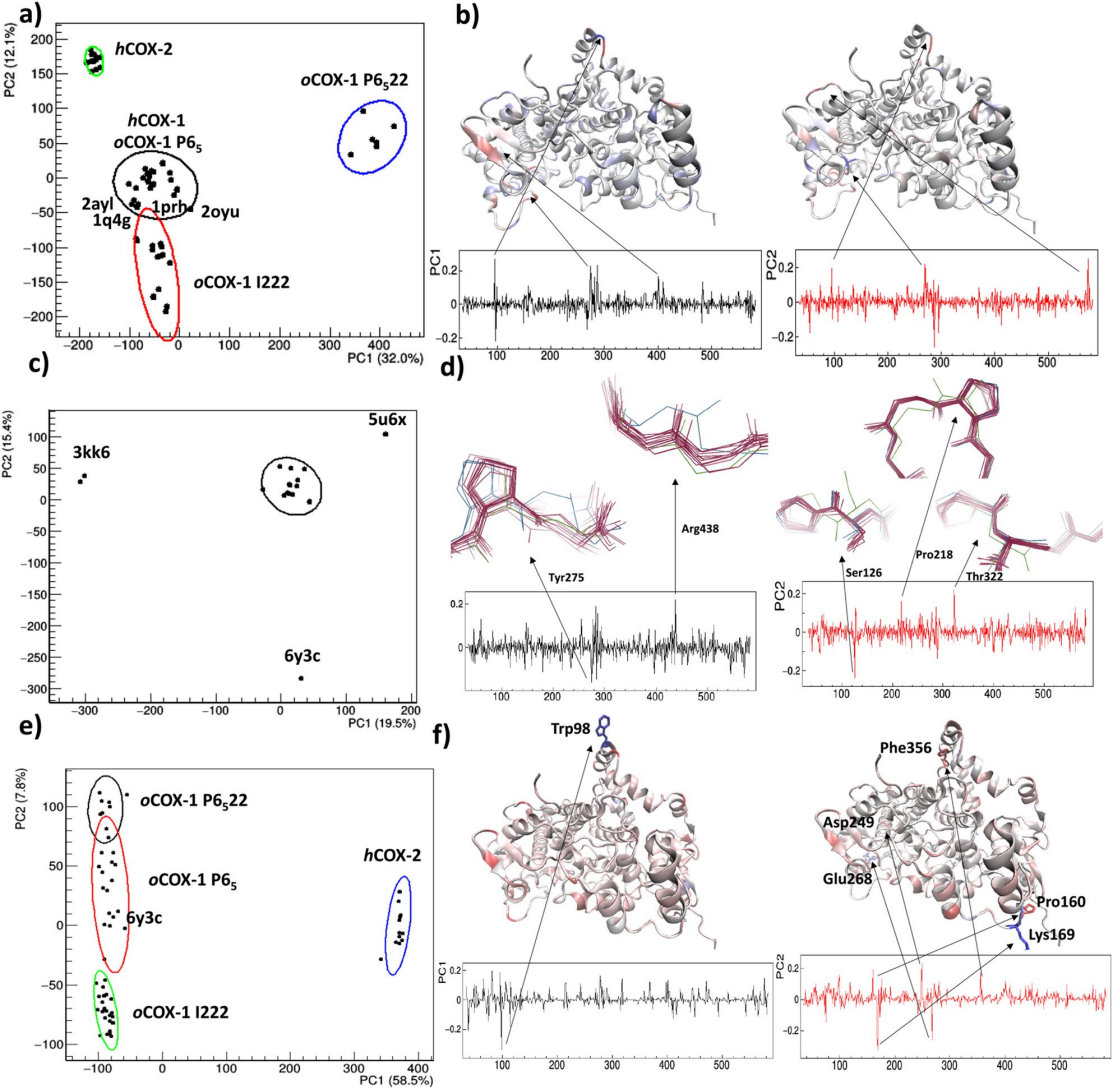
Comparison of *h*COX-1, *o*COX-1, and *h*COX-2 structural models by principal component analysis applied on PAV (**a**–**d**) and SASA (**e**,**f**) profiles. A restricted number of COX-1 structural models is considered in (**c**,**d**). [On the left is shown the score plot of the first two principal components, where representative points are grouped according to a hierarchic clustering algorithm. 85% confidence level ellipses are shown, and PDB codes associated with representative points are reported. The percentage of the total data variance explained by each principal component is shown on the axes. On the right are shown the loadings of the first (black) and second (red) principal components, the structural model 6Y3C colored according to the loading values, from red for higher positive to blue for higher negative (**b**,**f**), and the superposed structural models with 6Y3C and 3KK6 colored respectively in green and blue (**d**). Arrows point to protein residues having the highest and lowest loadings values. Residues falling in these regions are drawn in “bonds” representation in (**f**)].

In addition to the analysis based on Cartesian coordinates, we performed a comparative analysis based on backbone dihedral angles more focused on differences due to hinge motion. Profiles describing the residue-by-residue hinge flexibility have been calculated in terms of the Protein Angular Value (PAV). The 74 × 550 (chains × residues) matrix formed by the PAV profiles was processed by principal component analysis (PCA) to reduce the dimensionality of the problem and assess the separation among structural models in the 2D space defined by the first two principal components. Data points, representing structural models, can be grouped according to their position in the PC2 vs PC1 scores plot (Fig. 8a). We found that the chains belonging to the same structure have very close PC1/PC2 coordinates in the score plot to such an extent that the two chains cannot be distinguished. This result points out that each analyzed structure is made by two chains having the same hinge points, thus enforcing the hypothesis that their biological unit contains a homo-conformational dimer. Moreover, Fig. 8a shows that *h*COX-2 structures are consistently separated from COX-1 structures and are grouped in a very narrow cluster, confirming their higher structural homogeneity already observed by RMSD analysis. Instead, COX-1 structures exhibit a larger spread, with a huge separation of *o*COX-1 structures with P 6_5_22 symmetry, located at high positive PC1 values, and of *o*COX-1 structures with I 222 symmetry, located at high negative PC2 values. A cluster, including *o*COX-1 structures having symmetry P 6_5_, our *h*COX-1 structure and the outlier structures 2OYU, 1PRH, 2AYL, and 1Q4G is located at PC1 and PC2 values. Residues that are responsible for the above separation, as summarized in Table 3, can be identified among the highest values (positive or negative) of the PCA loadings (Fig. 8b). The effect of each individual range of hinge residues on structural separation has been determined by applying PCA on the PAV matrix restricted to that range. With this method, we were able to identify the residues 269–270 and 574–575 as main responsible for the separation of *h*COX-2 crystal structures, the residues 95–96 and 400–402 as responsible for the separation of *o*COX-1 P 6_5_22 structures and the region 274–287, which is able to separate *o*COX-1 structures with P 6_5_22 and I 222 symmetry. This latter region is interested in inter-molecular hydrogen bond interactions with the glycosylation site Asn410 in *o*COX-1 crystal structures (see Supplementary Table 1).

**Table 3:** Key residues determined by backbone dihedral angles comparative analysis applied on *h*COX-1, *o*COX-1, and *h*COX-2 crystal structures. In the table are reported: the residue number, the structural elements and the structural region where the residues are located, the principal components having high loadings values in the specific residue range (fourth column) and the crystal structures mainly separated by applying PCA to the specific residue range (last column).

Residue number	Structural element	Structural region	Principal components interested	Crystal structures mainly separated
95, 96	α_3_-α_4_	Substrate site	PC1, PC2	*o*COX-1 P6_5_22
269, 270	α_6_-α_7_ loop	Close to heme site	PC2	*h*COX-2
274, 281, 287	α_6_-α_7_ loop	Close to Asn410 Glycosylation site	PC1	*o*COX-1 P6_5_22, *o*COX-1 I222
400, 401, 402	β_12_	NAG site	PC1	*o*COX-1 P6_5_22
574, 575	C-term loop	C-term	PC2	*h*COX-2

A comparative structural analysis more focused on the cluster containing our *h*COX-1 structure has been carried out by removing from the selection the structures having higher deviations, i.e., *h*COX-2 and *o*COX-1 with P 6_5_22 and I 222 symmetry (Fig. 8c). By using this strategy, the *h*COX-1 (6Y3C) structure is separated from remaining *o*COX-1 crystal structures by PC2, while PC1 discriminates the 3KK6 chains, confirming that this latter structure is peculiar respect to the other *o*COX-1 with P 6_5_ symmetry, as found by RMSD analysis, and 5U6X, which was indeed used to drive the phasing of 6Y3C by molecular replacement. Loading analysis (Fig. 8d) allows identifying residues mainly responsible for the discrimination among selected structures: 438 and 275, which separates 3KK6 along PC1, and 322, 218 and 126, which separate 6Y3C along PC2. It should be noted that the conformational changes shown by the above residues in the discriminated crystal structures could reflect uncertainties due to limited data resolution. Thus, at this level of details, the discrimination is not significant, as confirmed by the limited data variability explained by the first two principal components (35%) in Fig. 8c. PCA applied to PAV appears able to distinguish crystal structures according to conformation changes, even if small, and, therefore, it represents a valuable diagnostic tool to check crystal structures against an ensemble of homologous crystal structures. For *h*COX-1, we verified that aligning the orientation of residues around Thr322, Pro218 and Ser126 to that shown by the P6_5_ *o*COX-1 chains does not lead to a better structural refinement against diffraction data.

Due to the nature of the crystal structure used as input for PAV analysis, such results rely on static information only. In an attempt to analyze flexibility from more dynamic information, we performed a coarse-grained modelling on our *h*COX-1 (6Y3C) and on the *o*COX-1 crystal structure having the highest resolution among those ones with the same space group of 6Y3C (2OYU) (Supplementary Discussion 2). The residue-by-residue Root Mean Squared Fluctuations (RMSF) values obtained by this modelling appear slightly higher in the case of *h*COX-1 (Supplementary Fig. 10a). By plotting RMSF values against Solvent Accessible Surface Area (SASA) values (Supplementary Fig. 10b), it is possible to observe that residues showing largest changes of flexibility between the two isoforms also show very large changes of SASA due to crystal contacts, but no differences for SASA due to dimer formation. This result suggests that residues characterized by largest differences in flexibility between the two isoforms are involved in crystal contacts, but do not interfere with dimer formation. In addition, it would provide a rationale to the fact that the two proteins have similar activity (related to dimer formation) and different crystallizability (related to crystal packing).

### Comparison based on COXs solvent accessibility

Conformational differences in protein structures usually result in significant differences in protein properties, also in the case of proteins with high homology or similar functions. Among these properties, solvent accessibility is of paramount importance because it determines the possibility of exogenous molecules to target protein pockets. RMSD and flexibility analysis suggested that our *h*COX-1 structure is comparable to *o*COX-1 structures and well separated with respect to *h*COX-2 structures. To assess how solvent accessibility discriminates COXs, we calculated the residue-by-residue SASA by scanning the protein surface with a probe of 1.4 Å of radius (water radius), and we performed PCA on SASA profiles, The resulting score plot (Fig. 8e) shows a clear separation between *h*COX-2 and *h*COX-1 structures along PC1, which explains 58.5% of the total data variance. According to the loading plots (Fig. 8f), the residue 98, which is a highly exposed tryptophan in COX-1 structures (SASA ≈ 200 Å^2^) and a glycine in COX-2 structures (SASA ≈ 50 Å^2^), is mainly responsible for this separation. Instead, COX-1 structures are separated along PC2, which explains only a small percentage of the total data variance (7.8%). *o*COX-1 crystal structures are grouped according to their crystal symmetry, with I 222 and P 6_5_22 structures located respectively at large negative and positive values of PC2 scores, and P 6_5_ structures positioned at small values of PC2 scores. As in the case of PAV analysis, our *h*COX-1 crystal structure is located in a peripheral region of the I 222 cluster of *o*COX-1 structures, pointing out slightly different solvent accessibility of this structure with respect to most of the other *o*COX-1 structures with same packing. Loadings analysis (Fig. 8f) clarifies that the solvent accessibility separation among COX-1 structures is due to the leading contribution of few differently exposed residues: Lys169 and Glu268, having the highest negative PC2 loadings values, hence responsible for the separation of the I 222 crystal structures, and Asp249, Pro160, and Phe356 , having the largest positive PC2 loadings values, hence responsible for the separation of the P 6_5_22 crystal structures. These residues are not mutated and do not assume particular conformations in *h*COX-1, so that it is not discriminated along PC2 (the 6y3c PC2 score value is nearly zero in Fig. 8e).

Highly exposed residues dominate structural comparison based on solvent accessibility, and in fact, all the above residues are found on the protein surface. However, it is interesting to perform a solvent accessibility analysis dedicated to two pockets that are important for COX activity, i.e., the substrate and heme sites. To this aim, SASA was calculated by increasing the probe radius from 1.4 to 6.0 Å for residues located in these sites and for three representative crystal structures: 6Y3C (*h*COX-1), 1U67 (*o*COX-1), and 5F19 (*h*COX-2). Such range of probe radius allowed to get information both from small (at low radius) and large (at high radius) solvent channels, thus providing a complete view of the accessibility of the two sites. SASA has been calculated after the removal of ligand molecules from protein structures. The ligand pocket volume can be characterized by the curves describing the SASA values averaged over the pocket residues as a function of the probe radius (Supplementary Fig. 12). For averaging, we consider only the contributes from conserved residues among proteins. In this way, SASA takes into account the steric hindrance of all residues but is not affected by the difference in the molecular surface of the residues exploited for averaging. The result of this analysis shows similar accessibility for *h*COX-1 and *o*COX-1. In the case of the substrate site, we found that the solvent accessibility of *h*COX-2 is higher than COX-1 proteins and decreases faster by increasing the probe radius. For the heme site, the accessibility of the three proteins is the same at 1.4 Å probe radius and, once again, it decreases faster for *h*COX-2. Therefore, also by restricting the analysis to the two pockets that are important for COX activity, we found similarity between *h*COX-1 and *o*COX-1. Interestingly, SASA decreases significantly between 1.4 and 2.5 Å probe radius regardless of protein sequence, and such a decrease is more pronounced in the case of *h*COX-2.

In the case of the heme site, SASA can be ascribed to a different steric hindrance due to residue variations rather than to different conformations. Indeed, the SASA analysis performed on protein molecules converted in polyalanine chains, so that the effect of side chains is excluded from calculations (Supplementary Fig. 13), provides the same accessibility for each of the three protein molecules. Instead, in the case of the substrate site, accessibility appears to be related to different conformations, because the SASA plot obtained for polyalanine-converted protein molecules has the same features.

**Figure 9:**
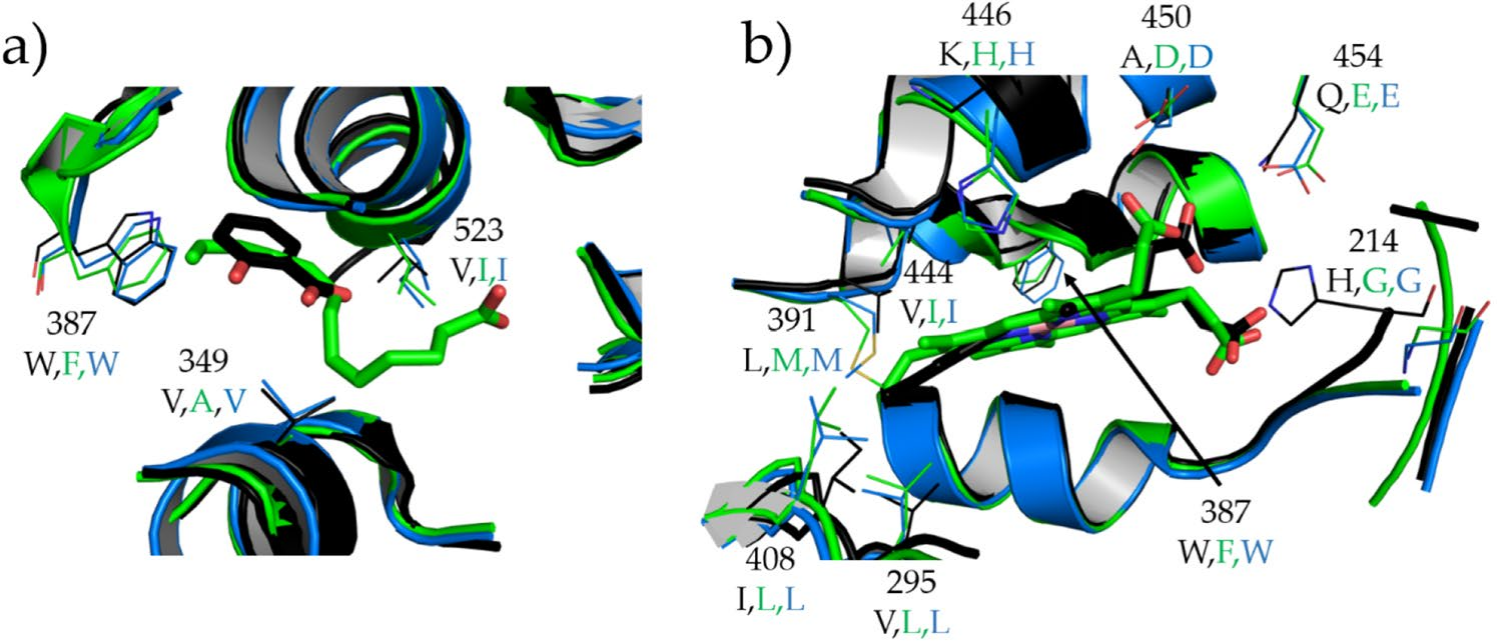
Comparison of the substrate (**a**) and heme (**b**) sites of COX proteins. *h*COX-1 (6Y3C) is in cyan color, *o*COX-1 (1U67) in green color, and *h*COX-2 (5F19) in black color. Residues not conserved among the proteins are drawn in line representation, whereas ligand molecules related to *o*COX-1 and *h*COX-2 are drawn in stick representation.

A detailed view of the two pockets (Fig. 9) can be used to interpret the above analysis result. In the case of the heme site (Fig. 9b), *h*COX-1 and *o*COX-1 have the same residues, which explains the similar trend of their solvent accessibility. The faster drop of SASA curves of *h*COX-2 can be explained by the presence of residues that are bulkier in the *h*COX-2 than COX-1 proteins (particularly for 387, 446, and 214). In the case of the substrate site (Fig. 9a), *h*COX-1 and *o*COX-1 have different residues, but these variations do not affect their SASA trends, which are still similar.

### Comparison of glycosylation sites

Our crystal structure clearly shows the same glycosylation site of *o*COX-1 and *h*COX-2 (asparagine residues 68, 144, and 410). To compare intra- and inter-molecular hydrogen bond interactions occurring in these glycosylation sites, the crystal structures 2OYU, 1Q4G, 4O1Z, and 5F19 have been considered, as they have the highest data resolution among the *o*COX-1 crystallized in the P 6522, I222, P 65 space group, and the *h*COX-2 crystallized in the I 222 space group, respectively. Inter- and intra-molecular interactions are shown in Supplementary Tables 1 and 2, respectively. Five intra-molecular hydrogen bonds are conserved among the proteins under investigation: Asn144-Ser146, Asn410-Gln406, Asn68-Gln42, Asn68-Tyr55, and Asn410-Leu408. The last interaction is conserved in *h*COX-2, despite Leu408 is mutated in Ile. In addition, *h*COX-1 and most of the *o*COX-1 crystal structures share the interactions Asn144-Glu140**,** Asn410-Ser412, Asn410-Met413, and Asn68-Pro67. In the case of *h*COX-2, Asn410-Ser412 occurs at a distance slightly higher than that considered for our comparison (3.2 Å), while the other two interactions are likely lost due to the mutations of Met413 and Pro67 in Ile and Glu residues, respectively. Instead, Asn410-Asp416 appears conserved in *o*COX-1 crystallized in P 6_5_ space group (4O1Z) and in *h*COX-2, where Asp416 is mutated in Glu. Importantly, we do not found interactions neither present nor absent only in *h*COX-1.

On the contrary, inter-molecular hydrogen bonds do not involve glycosylation sites in *h*COX-1, differently from *o*COX-1 and *h*COX-2. For these two proteins, we found that the glycosylation on Asn144 plays an important role in dimer formation (Supplementary Fig. 11a). Asn144 interacts with Glu239, which is mutated in alanine in *h*COX-2. Here, the interaction is formed with Leu238 that, interestingly, appears to shift the α-helix 237–245 much more than the Asn144-Glu239 interaction does in *o*COX-1. The glycosylation site on Asn410 is involved in inter-molecular hydrogen bonds only in *o*COX-1 crystal structures. Particularly, Asn410-Gln282 characterizes *o*COX-1 crystallized in the I222 space group (1Q4G), and Asn410-Gly278 characterizes those crystallized in the P 6_5_22 and P 6_5_ space groups (2OYU and 4O1Z) (Supplementary Fig. 11b-d). It is noteworthy that in the case of 2OYU and 4O1Z, the position of the amidic carbon atom of the *N*-acetylglucosamine bound to Asn410 should be inverted with that of the oxygen atom of the same group to allow the interaction between this glycosylation and Gly278. Finally, glycosylation on Asn68 interacts with Asp584 and Leu176 in the case of *o*COX-1 crystallized in P_65_ (4O1Z) and *h*COX-2 (5F19), respectively (Supplementary Fig. 11e-f).

## Discussion

For decades, *o*COX-1 has been exploited as a surrogate for the human enzyme, which is more challenging to produce in milligram-quantity and recalcitrant to crystallization^13^. In this paper, we report the first three-dimensional crystal structure of *h*COX-1 along with an extensive structural comparison with known crystal structures of the constitutive (COX-1) and inducible (COX-2) isoforms of the prostaglandin-endoperoxide H synthase. *h*COX-1 has a very high sequence identity with *o*COX-1 (92%) and, like the ovine enzyme, also crystallizes in a hexagonal space group in the presence of lithium chloride and sodium citrate^19,28^. The remaining crystallization cocktail exploits PEG 4 K as a precipitant and leads to an orthorhombic unit cell, in which protein molecules are more tightly packed than those in the hexagonal cell (the solvent content is ≈ 72% and ≈ 65% for orthorhombic and hexagonal unit cell, respectively), likely due to the lower ionic strength of the crystallization cocktail. A comprehensive analysis of the diffraction-data precision indicator (DPI) among known COX crystal structures (Supplementary Fig. 6) pointed out the lower crystalline order of *h*COX-1 with respect to *o*COX-1 s and *h*COX-2 s.

In the comparative analysis of the *h*COX-1 crystal structure with those of the ovine enzyme and *h*COX-2, we considered different types of variables connected to crystal structures: (1) Cartesian coordinates, aiming at identifying specific regions showing conformations changes, (2) backbone dihedral angles, aiming at locating hinge points responsible for variations in protein flexibility, (3) solvent accessibility, aiming at finding differently exposed residues. All the analyses agree in confirming that conformationally equivalent protein units make the biological unit of *h*COX-1, *o*COX-1 and *h*COX-2 crystal structures. We delineated two regions characterized by positional deviations between *h*COX-1 and all the other structures investigated: the α_6_-α_7_ loop, comprising residues from 270 to 290, and the region 70–110, covering the first two α-helices of the membrane-binding domain. The first region is a key region for determining separations among crystal structures due to its intrinsic flexibility, which is influenced by changes in sequence or symmetry. Here, no significant differences have been observed between *o*COX-1 and *h*COX-2 with respect to our *h*COX-1 by observing ligand interactions and interactions involving glycosylation sites. The second region is mainly affected by crystal packing variations (orthorhombic *versus* hexagonal). Such region shows differences in ligand interaction between *h*COX-1 and *o*COX-2 crystallized in I 222 and difference in interactions involving the glycosylation sites Asn68 of *h*COX-1 and *h*COX-2. Other regions characterized by large deviations emerged from the comparison of our *h*COX-1 structure with *h*COX-2 ones and could be explained by the differences between the interaction involving the glycosylation sites Asn144 and Asn410 of these two proteins.

The analysis based on backbone dihedral angles confirmed the key role played by the above regions to determine separations among COX-1 and COX-2 structures, as specific hinge residues have been systematically found within them (Supplementary Table 3). In other words, we have determined the residues that, changing their orientation, induce large variations of the position of nearby residues, which means that detected differences in the relative position of protein regions in different structures can be attributed to hinge motion. For example, within the region 270–290 we were able to identify residues 274, 281 and 287 as primarily responsible for the separation of *o*COX-1 structures with P 6_5_22 symmetry, and residues 269, 170 and 287 as responsible for the separation of *h*COX-2 structures and *o*COX-1 structures with I 222 symmetry (Table 3). Residues 95 and 96 are, instead, hinge points that determine unique conformations of the 70–110 region in P 6_5_22 *o*COX-1 structures and in 1PRH (Table 3). Additional separation of *h*COX-2 structures is also characterized by unique conformations of their C-term loop, determined by changes in orientations of the hinge residues 574 and 575 (Table 3).

A flexibility analysis performed on similar COX-1 structures highlighted residues showing conformational changes, which are responsible for the discrimination of few outlier crystal structures. In particular, peculiar conformations of Thr322, Pro218, and Ser126 were found in our *h*COX-1 crystal structure when compared with those of the P6_5_ *o*COX-1 crystal structures. It is not clear if such differences are originated by variations in the primary structure or are an effect of the limited data resolution. In this respect, the combined PAV-PCA analysis has shown its potentiality in pinpointing divergences in the orientation of residues within an ensemble of homologous crystal structures.

The comparative analysis based on solvent accessibility highlighted the role of residue 98 in discriminating *h*COX-2 structures due to the huge change in its side chain going from *h*COX-2 to *h*COX-1/*o*COX-1 sequence (Gly to Trp). This residue still falls in the region 70–110 emerged from the RMSD and dihedral angle analyses. A study dedicated to characterizing the solvent accessibility of the substrate and heme sites has revealed quite a similar shape of these cavities in *h*COX-1 and *o*COX-1 crystal structures.

**Figure 10:**
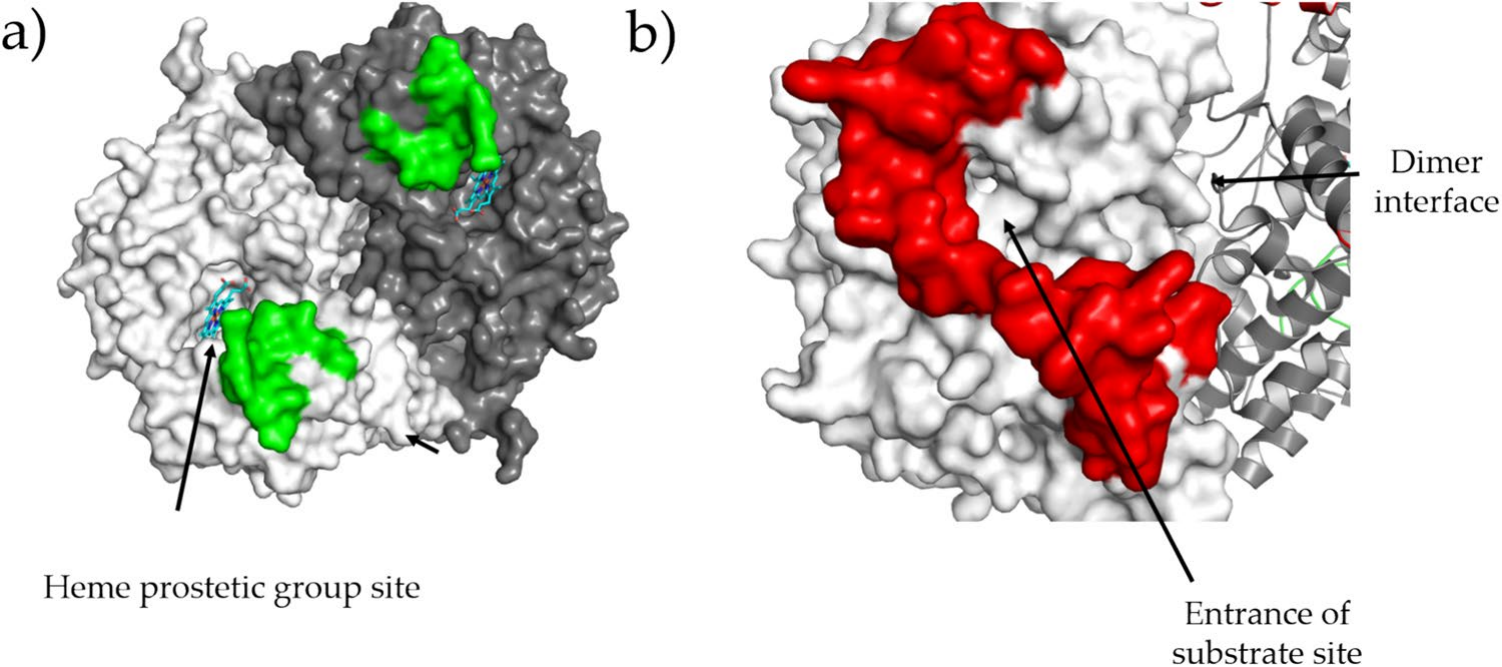
Regions whose conformation is peculiar for *h*COX-1. Protein units forming the dimer are shown in white and gray surface. (**a**) The heme molecule is shown in stick representation and the region 270–290 in green color. (**b**) The region 60–100 is shown in red color.

The two regions whose conformation is peculiar for our *h*COX-1 are shown in Fig. 10. The region between residue 270–290 (Fig. 10a) is part of the loop surrounding the heme site and does not appear directly involved in the electron-transfer mechanism occurring at this site between prostaglandin G_2_ (PGG_2_) and prosthetic group; however, by considering its position on the protein surface, a role of this region in the binding mechanism of PGG_2_ to COX-1 can not be excluded. The region 70–110 (Fig. 10b, red color) links EGF to the membrane-binding domain and, therefore, is close to the substrate pocket. Picot et al. divide such a pocket into two regions named lobby and active site, both important for inhibitor binding^1,5,28^. The lobby site is very close to the region between residues 70 and 110; therefore, differences in the conformation of this region are expected to affect the lobby region, thus the inhibitor binding mechanism.

In summary, the crystal structure of *h*COX-1 described in this paper represents a significant achievement in structural biology as well as medicinal chemistry, which paves the way for the development of new highly selective inhibitors^29–32^. Co-crystallization studies with substrate molecules and small-molecule inhibitors will be instrumental in clarifying the unique biochemical properties of the human enzyme. Future studies that build upon the structure presented here will help in the development of novel therapeutics and imaging-probe that target *h*COX-1 for unmet clinical diagnosis of diseases in which *h*COX-1 is a biomarker^9,10^.

## Materials and methods

Cell culture reagents were purchased from EuroClone (Milan, Italy). Protoporphirin IX (Hemin), trizma base, potassium chloride, imidazole, sodium citrate tribasic dihydrate and lithium chloride, Amicon Ultra-4 10 kDa MWCO centrifugal filter device was purchased from Merck. Nickel-NTA agarose beads (low density), *n*-octyl β-D-glucopyranoside (β-OG) and phenylmethylsulfonyl fluoride (PMSF) were from Gold Biotechnology. Hexaethylene Glycol Monodecyl Ether (C_10_E_6_) detergent was purchased from Anatrace (Maumee, OH). Phusion High Fidelity (*HF*) DNA was from Biolabs. Sf-900 II SFM medium, HALT protease inhibitor single-use cocktail EDTA-free, Pierce BCA protein assay kit, Snakeskin dialysis tubing (10 kDa MWCO, 35 mm), and glycerol were purchased from Thermo Fisher Scientific Italia (Monza, Italy). TGX 10% precast polyacrylamide gels and all reagents for SDS-PAGE were purchased from BIO-RAD Laboratories Srl (Milan, Italy). Tobacco Etch Virus (TEV) protease was produced as described^33^. Oxygraph buffer: 200 mM Tris–HCl (pH 8.3), 2 M KCl, 1 mM phenol.

### Construction of the baculovirus recombinant expression vector, protein production and purification

Cloning into baculovirus DNA of *h*COX-1 cDNA bearing an import signal peptide followed by an N-terminal 8X-His tag and a TEV cleavage was carried out as follows. Briefly, *h*COX-1 cDNA was obtained by PCR using Phusion High Fidelity (HF) DNA polymerase and two specific primers For and Rev bearing at their 5′-end a BglII and a NotI site, respectively. Primer sequences (5′ to 3′) are: PBac.*h*COX-1.BglII.For: CACAAGATCTGATATGAGCCGGAGTCTCTT and PBac.*h*COX-1.NotI.Rev: CACAGCGGCCGCTCAGAGCTCTGTGGATG. DNA template was a recombinant pFastBac1/*h*COX-1 cDNA gently provided by Prof. William Smith (Michigan University). The PCR reaction was carried out in a 50 µl volume, using 3 ng of DNA, 0.2 mM dNTPs, 0.5 μM for each primer, 1X Phusion HF buffer containing MgCl_2_ and 1 U of Phusion High-Fidelity DNA Polymerase (BioLabs). After heating at 98 °C for 40 s, the reaction was subjected to 30 cycles consisting of 98 °C for 30 s, 68 °C for 1 min and 72 °C for 40 s; the final elongation was performed at 72 °C for 7 min. The amplification product was purified using Eurogold Cycle Pure kit (Euroclone) according to the manual instructions, and quantified by measuring the absorbance at 260 nm with the NanoDrop 1000 (Euroclone); it was then restriction-digested with BglII and NotI, purified, quantified as before, and finally inserted into the BglII/NotI sites of the pBacPAK9 baculovirus shuttle vector (Clontech), using standard DNA manipulation techniques. For DNA ligation the Rapid DNA Ligation Kit (Roche) was used according to the manufacturer’s protocol, to yield the pBacPAK9/*h*COX-1 recombinant vector. Ligation mixture was transformed into XL1-blue competent E. coli cells according to the standard protocol employing CaCl_2_. Positive clones were identified by restriction digestion of the miniprep DNA; the correct sequence of the insert was verified by automatic sequencing of both strands (Eurofins Genomics Italy). pBacPAK9/*h*COX-1 plasmid and linearized baculovirus DNA (BacPAK6 Viral DNA Bsu36 I digest) were co-transfected in *Spodoptera frugiperda* (Sf9) cells according to the manufacturer's protocol^34^. The co-transfection supernatant was subjected to plaque assay to obtain individual viral plaques; four single and well separated agarose plaques corresponding to pure viruses were picked and immersed into 500 μl of Sf-900II SFM (Gibco) with 1% penicillin/streptomycin (P/S) (Gibco) and 5% fetal bovine serum (FBS) (Gibco) for 16 h at + 4 °C in the dark, to allow viruses to diffuse out of the agarose. Putative recombinant viruses from each plaque were tested for the presence of *h*COX-1 insert; for that, each plaque supernatant was subjected to a one-step amplification, yielding Virus Stock I (VSI), from which the DNA viral was extracted according to McCarthy CB et al. with some modifications^15^. A 200 μl volume of four VSI was lysed by gentle mixing with an equal volume of Lysis Buffer (100 mM Tris–HCl pH 7.6, 10 mM EDTA, 0,25% SDS) followed by a 10-min incubation at room temperature. Samples were extracted once with phenol/chloroform/isoamyl alcohol and once with chloroform/isoamyl alcohol; the nucleic acids were ethanol precipitated, dissolved in water and quantified spectrophotometrically. A 100 ng amount of viral DNA was analyzed by PCR in a final volume of 50 μl, using Taq Polymerase TaKara (Clontech) (2.5 U), dNTPs 0.2 mM, 1 μM of each specific primer For and Rev used for cloning (see above). After heating at 98 °C for 15 min, the reaction was subjected to 30 cycles of 98 °C for 30 s, 52 °C for 1 min and 72 °C for 2 min, followed by an extension at 72 °C for 5 min. All of the four analyzed plaques resulted positive. One VSI was chosen, that was amplified twice yielding VSII and VSIII; the latter was titred by plaque assay (~ 5 × 10^8^ pfu/ml) and used as inoculum stock for recombinant protein expression.

Sf9 insect cell culture suspension at a cell density of 2 × 10^6^ cells/ml in 4 L of the Sf-900 II SFM medium (5% FBS, 1% P/S) was infected with the recombinant virus stock VSIII at a multiplicity of infection (pfu/cell) of 10. After 72 h, infected Sf9 cells were harvested by centrifugation at 900xg at + 4 °C for 20 min. The cell pellet was rapidly cooled in liquid nitrogen and stored at − 80 °C until use. The following steps were performed at + 4 °C. For 8xHis-tagged *h*COX-1 purification, cell pellet was resuspended in 30 ml of cold lysis buffer [20 mM Tris–HCl pH 8.0, 100 mM KCl, containing Complete EDTA-free protease inhibitor (Roche) and 1 mM PMSF] and disrupted by sonication (7 microtip of potency, 1″ pulse on and 4″ pulse off for 5 min, sonicator Branson) on ice (*ovine* or *human* COX-1/Sf9 sonicated pellet). C_10_E_6_ detergent was added (1.12% w/v) and solubilization was performed with gentle shaking at + 4 °C overnight (o/n). The lysate was clarified by centrifugation at + 4 °C for 75 min at 62,000×g , and the resulting supernatant was incubated with Ni^2+^-NTA agarose beads (low density, 50% slurry) pre-equilibrated twice with 3 column volumes of *Ni*-Buffer [20 mM Tris pH 8.0, 100 mM KCl, 5 mM imidazole, 0.1% (w/v) C_10_E_6_, 5% glycerol] and rocked for 3 h at + 4 °C. One milliliter of Ni^2+^-NTA agarose resin binds 5 to 10 mg of 8X-His fusion protein. The protein-resin slurry was poured into the chromatography column, and the flowthrough (FT) was collected slowly. The beads were washed sequentially with ten-bed volumes of pre-chilled wash buffer I [20 mM Tris–HCl, pH 8.0, 500 mM KCl, 10 mM imidazole, 0.1% (w/v) C_10_E_6_, 5% glycerol] and five-bed volumes of wash buffer II [20 mM Tris–HCl pH 8.0, 40 mM KCl, 20 mM imidazole, 0.1% (w/v) C_10_E_6_, 5% glycerol]. Finally, the His-tagged *h*COX-1 protein was eluted with 1–3 bed-volumes of elution buffer [20 mM Tris–HCl pH 8.0, 40 mM KCl, 250 mM Imidazole, 0.1% (w/v) C_10_E_6_, 5% glycerol]. The FT fraction was subjected to a further chromatography step by re-incubating it with the resin in batch with gentle rotation, o/n. The *h*COX-1 eluted fractions were combined and dialyzed o/n against 500 ml of no-imidazole buffer [20 mM Tris–HCl pH 8.0, 150 mM KCl, 0.1% (w/v) C_10_E_6_, 5% glycerol]. Histidine tag was cleaved off with TEV_6His_ protease at 1∶40 (w/w) protease to protein ratio. After digestion, the reaction mixture was passed on fresh Ni-beads to recapture the protease^16^. *h*COX-1 was recovered in the FT and beads were washed with buffer without imidazole [20 mM Tris–HCl pH 8.0, 100 mM KCl, 0.1% (w/v) C_10_E_6_, 5% glycerol]. Protein purity was assessed by SDS-PAGE analysis followed by staining with Coomassie Brilliant Blue G-250. Protein concentration was assessed spectrophotometrically by NanoDrop 2000 (Thermo Scientific) measuring the absorbance at 280 nm using the theoretical molar extinction coefficient (96,720 M^−1^ cm^−1^) determined from the amino acid sequence and molecular weight to 70 kDa.

### Cyclooxygenase activity evaluation by O_2_ measurement-based assay

Functional analysis of *ovine* and *human* COX-1 catalytic activity was determined by monitoring O_2_ consumption by using an Oxytherm electrode unit (Hansatech), in the presence of arachidonic acid and different inhibitors. The instrument is equipped with a Clark-type oxygen electrode to monitor the dissolved oxygen concentration in a sealed measurement chamber over the time. Oxygen consumption was measured at 37 °C directly in a 1000 µL reaction vessel. Briefly, it follows the description of a typical assay: Oxygraph buffer (the opportune volume is adjusted to 1000 µL of final volume according to the volume of the protein), 5 µM hemin (1 µL), 100 µM arachidonic acid (20 µL) and inhibitor (10 µL) at the opportune concentrations. By this feasible approach, COX-inhibitory activity (IC_50_) of known NSAIDs such as mofezolac, ibuprofen and indomethacin was evaluated. Mofezolac was chosen as an example of highly selective COX-1 inhibitor, while indomethacin and ibuprofen as non-selective COXs inhibitors.

Oxygen consumption determination (μmol/ml O_2_) started after the addition of the ovine or human COX-1/Sf9 sonicated pellet (C = 2.6 mg/ml) to the mixture containing Oxygraph buffer, arachidonic acid and test compounds at the opportune concentrations. Assay mixture was allowed to equilibrate in the reaction vessel at 37 °C for several minutes until steady baseline was recorded. Uninfected Sf9 sonicated cells were used as a negative control. Stock solutions were prepared in a minimum volume of vehicle (DMSO).

Data were collected by a PC and analyzed by an Oxygraph Plus Software. The change in O_2_ concentration, expressed as l micromole of oxygen per minute at 37 °C (O_2_ consumed/min) were obtained from the first derivative of [O_2_] versus time.

### Protein crystallization, data collection, and structure determination

*h*COX-1 was buffer exchanged against 20 mM HEPES at pH 7.3, 40 mM NaCl and 0.4% w/v β-octyl glucoside using an Amicon Ultra 4 ml concentrator (with membrane Ultracel-PL PLGC, MWCO 10 kDa) and concentrated to 15 mg/ml. As for COX-2, also COX-1 requires heme (Fe^3+^–protoporphyrin IX) as a cofactor. Therefore, 1 mM protoporphyrin in DMSO was added to the protein solution in a 1:1 molar ratio. The solution was incubated at 4 °C for 10 min and used for crystallization. Protein crystals were obtained in 2 weeks by using the sitting-drop vapor diffusion method at 293 °K by mixing 1.5 μL of protein solution with 1.5 μL of reservoir solution consisting of sodium citrate 0.7 and 0.58 M LiCl. The pH value of the reservoir solution was not adjusted. Crystallization experiments were performed at 10.4 mg/ml of protein concentration based on the previous optimization done using the Hampton Research Pre-Crystallization Test. Crystals were flash-frozen in liquid nitrogen after a quick dipping in a solution containing reservoir solution supplemented with 20% glycerol as cryoprotectant. X-ray diffraction data collection was performed at the I04 beamline of Diamond Light Source Ltd Synchrotron (Didcot, Oxfordshire, UK) by using 0.9795 Å as wavelength. Data reduction was performed by XIA2^35^ in 3dii mode, thus using XDS^36^ for indexing, scaling and merging. TRUNCATE^37^ included in CCP4 crystallographic suite^38^ was used to convert reflection intensities to amplitudes and to select reflections for R_free_ calculation. Data have been processed by STARANISO^39^ to remove anisotropy. The REMO program^40^ included in the package SIR2014^41^ was used to solve the structure by Molecular Replacement. Here, the crystallographic structure of ovine COX-1 (PDB code 5U6X)^13^, which has 92% of sequence identity with respect to the *h*COX-1, was used as a starting model. Structural refinement was performed by using Phenix.refine^42^ included in the crystallographic suite PHENIX^43^. Once refined, the *h*COX-1 crystal structure has been validated using the PDB validation server^44^ and deposited in the Protein DataBank with accession code 6Y3C. PISA server^20^ was used to define *h*COX-1 biological unit and PRO-ORIGAMI^45^ along with TOPDRAW^46^ to determine and draw the protein topology*.*

### Comparative structural analysis

The *h*COX-1 crystal structure was compared with known COX-1 from *ovis aries (*found by setting a search query in the PDB based on the sequence of *o*COX-1 having UniProt code P05979, identity E-value = 10^6^ and cutoff identity = 95%) and *h*COX-2 structures (found by setting a search query in the PDB based on the sequence of *h*COX-2 having UniProt code P35354, identity E-value = 10^6^, and cutoff identity = 95%) by using residue-by-residue, Solvent Accessibility Surface Area (SASA) values, Cartesian coordinates of the Cα atoms and backbone dihedral angles. The first ones were calculated by the program AREAIMOL^47^ included in the crystallographic suit CCP4, the second ones were used to calculate the root mean square deviation (RMSD) between pair of structures, though the program SUPERPOSE^48^, the third ones were used to calculate the Protein Angular Value (PAV) associated to each residue^49^. PAV is defined as:

1 $$PAV_{i} = \frac{180}{\pi }cos^{ - 1} \left( {\cos (\psi_{i} + \varphi_{i} )} \right)$$

where $\psi_{i}$ and $\phi_{i}$ are the backbone dihedral angles of the *i*-th residue. PAV values range between 0° and 180° and represent the $\psi_{i} + \phi_{i}$ values expressed in degrees, avoiding the problem of range definition connected with the circular nature of the angular variables. PAV profiles of each structure were calculated through the script TPAD^50^ run on VMD^51^ SASA, RMSD, and PAV profiles from different structures were separately analyzed by using Principal Component Analysis (PCA) and hierarchic clustering implemented in the program RootProf^52^. Crystal structures have been compared by considering individual chains of the biological unit. Solvent molecules, metal ions and ligands were removed from the PDB files, and protein residues between 34 and 583 were considered, as terminal residues were missing in some structural models. *h*COX-2 and *o*COX-1 sequences were aligned with the *h*COX-1 one, so that to consider 550 residues regardless of the type of protein. PCA was performed by considering two chains for each crystal structure to ensure a uniform weighting. In the case of SASA, residues having missing side chain in the final model of at least one crystal structure are excluded from PCA. The coordinate errors of the structural models used for comparative analysis were estimated by the diffraction-data precision indicator^53^, defined by:

2 $$DPI = \sqrt {\frac{{N_{atoms} }}{{N_{obs} }}} C^{{ - {\raise0.7ex\hbox{$1$} \!\mathord{\left/ {\vphantom {1 3}}\right.\kern-\nulldelimiterspace} \!\lower0.7ex\hbox{$3$}}}} d_{min} R_{free}$$

where $N_{atoms}$ is the number of atoms in the model, $N_{obs}$ is the number of independent reflections, *C* is the fractional data completeness, $R_{free}$ is the crystallographic R-factor and $d_{min}$ is the data resolution. DPI has been calculated by using the program SFCHECK^54^ when diffraction data are available, or by using Eq. (2) with information given in the PDB^55–65^.

## Supplementary Information

Supplementary Information 1.
