# Supplementary material for: Three-dimensional structure of human cyclooxygenase (hCOX)-1
Source: Sci Rep. 2021 Feb 22;11:4312. doi: 10.1038/s41598-021-83438-z (PMC7900114; doi:10.1038/s41598-021-83438-z)
Supplement: Supplementary file 1 — Supplementary Information 1. [file 41598_2021_83438_MOESM1_ESM.doc]

**Supplementary material to**

**“Three-dimensional structure of human cyclooxygenase (*h*COX)-1”**

Morena Miciaccia^1a^, Benny Danilo Belviso^2a^, Mariaclara Iaselli^1^, Gino Cingolani^3^, Savina Ferorelli^1^, Marianna Cappellari^4^, Paola Loguercio Polosa^4^, Maria Grazia Perrone^1^, Rocco Caliandro^2*^ & Antonio Scilimati^1*^

^1^Department of Pharmacy - Pharmaceutical Sciences, University of Bari "Aldo Moro", Via E. Orabona 4, 70125, Bari (Italy)

^2^Istituto di Cristallografia, Consiglio Nazionale delle Ricerche, via Amendola 122/o, 70126, Bari (Italy)

^3^Department of Biochemistry and Molecular Biology, Thomas Jefferson University, 1020 Locust Street Philadelphia, PA 19107 (USA)

^4^Department of Biosciences, Biotechnologies, and Biopharmaceutics, University of Bari, Via E. Orabona 4, 70125 Bari, Italy

^a^These authors contributed equally: Morena Miciaccia, Benny Danilo Belviso.

*****Corresponding authors:

Antonio Scilimati (antonio.scilimati@uniba.it)

Rocco Caliandro (rocco.caliandro@ic.cnr.it)

**Content:**

**Supplementary Fig. 1.** **PCR amplification of *h*COX-1 cDNA from pFastBac recombinant plasmid**.

**Supplementary Fig. 2. Restriction digestion analysis of miniprep DNA**.

**Supplementary Fig. 3. PCR analysis of viral clones (VSI).**

**Supplementary Fig. 4**. **Affinity purification of recombinant 8xHis *h*COX-1 expressed in insect cell.**

**Supplementary Fig. 5. Diffraction-data precision indicator (DPI) of the structural models used for comparative analysis**.

**Supplementary Fig. 6. Projection of the matrix of RMSD for 65 chains of COX-1 and COX-2 structures**.

**Supplementary Fig. 7. Residue-by-residue root mean square deviation (RMSD) values calculated after superposition of inter-cluster (left) and intra-cluster (right) representative crystal structures**.

**Supplementary Fig. 8. Interactions between ligands and region 70-110 of COX proteins.**

**Supplementary Fig. 9. Analysis of protein flexibility.**

**Supplementary Fig. 10. Inter-molecular interactions involving glycosylation sites.**

**Supplementary Fig. 11. SASA values averaged on conserved residues of the substrate (a) and heme (b) sites**.

**Supplementary Fig. 12. Solvent accessibility of COXs.**

**Supplementary Table 1. Inter- molecular hydrogen bond interactions with glycosylation sites Asn68, Asn144, and Asn410.**

**Supplementary Table 2. Intra-molecular hydrogen bond interactions with glycosylation sites Asn68, Asn144, and Asn410.**

**Supplementary Table 3. Correspondence between hinge points found by comparative analysis based on backbone dihedral angles and protein regions showing positional deviations highlighted by comparative analysis based on Cartesian coordinates**.

**Supplementary Discussion 1. Interactions between ligands and region 70-110 of COX proteins.**

**Supplementary Discussion 2. Bioinformatics analysis on COX-1 crystallizability.**


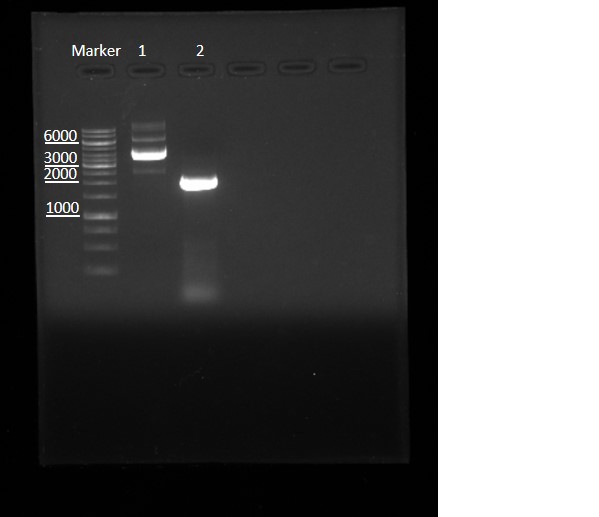


**Supplementary Fig. 1.** **PCR amplification of *h*COX-1 cDNA from pFastBac recombinant plasmid**. Amplification was performed by using Phusion HF DNA polymerase and specific primers. PCR products were analyzed on 1% agarose gel with ethidium bromide staining. M: GeneRuler 1 Kb Ladder (Thermo). Lane 1: Undigested product of pFastBac/*h*COX-1. Lane 2: PCR amplification product of pFastBac/*h*COX-1. The image is a full-length gel, and it conforms to the original gel.


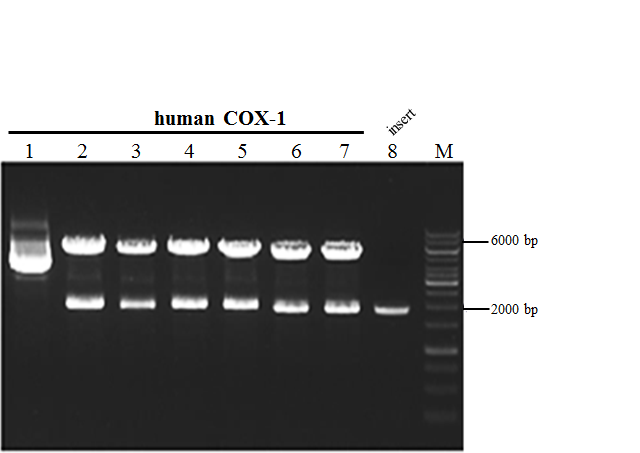


**Supplementary Fig. 2. Restriction digestion analysis of miniprep DNA**. DNA was extracted from several bacterial colonies and screened by restriction digestion with BglII and NotI enzymes. DNA was separated by gel electrophoresis on 1% agarose with ethidium bromide staining. Lane 1: undigested DNA; Lanes 2-7: digested DNA; Lane 8: *h*COX-1 PCR product (insert); M: GeneRuler 1 Kb Ladder (Thermo). Digestion of positive clones produced two fragments: the insert of about 2000 bp and the remaining vector of 6000 bp. The image is a full-length gel, and it conforms to the original gel.


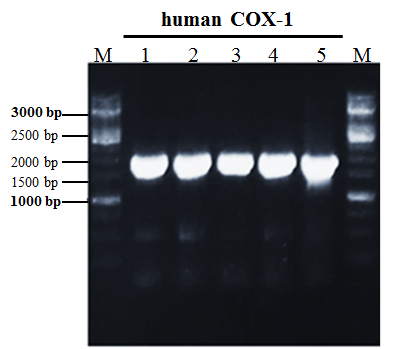


**Supplementary Fig. 3. PCR analysis of viral clones (VSI).** Viral DNA was extracted from VSI and subjected to PCR as described in the text. Products were analyzed on 1% agarose gel with ethidium bromide staining. PCR amplification of VSI DNA (lanes 1-4) and of pBacPAK9/*h*COX-1 as positive control (lane 5). M: GeneRuler 1 Kb Ladder (Thermo). The image is a full-length gel, and it conforms to the original gel.


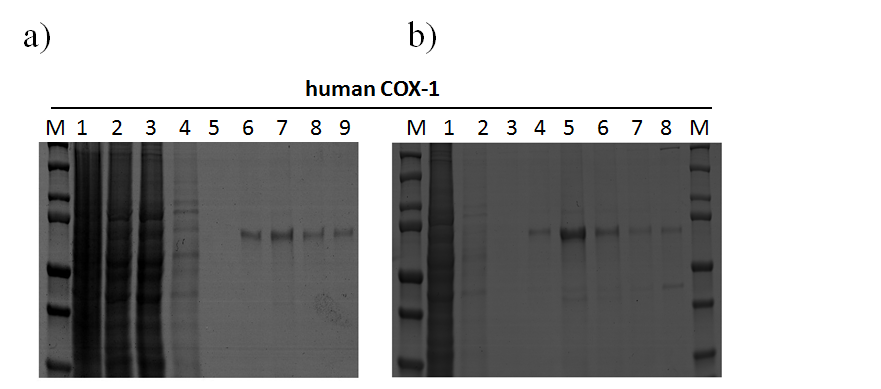


**Supplementary Fig. 4**. **Affinity purification of recombinant 8xHis *h*COX-1 expressed in insect cell.** The soluble portion (supernatant) of insect cell lysate was purified in batch using Ni^2+^-NTA resin and resulting fractions were analyzed by SDS-PAGE. a) M: Precision Plus Protein™ Dual Color Standards (Bio-Rad Laboratories); Lane 1: Solubilized lysate; Lane 2: Supernatant; Lane 3: FT; Lane 4: Wash with Buffer I (10 mM imidazole); Lane 5: Wash with Buffer II (20 mM imidazole); Lane 6-9: eluted fractions at 250 mM imidazole. b) M: Precision Plus Protein™ Dual Color Standards (Bio-Rad Laboratories); Lane 1: FT; Lane 2: Wash with Buffer I (10 mM imidazole); Lane 3: Wash with Buffer II (20 mM imidazole); Lane 4-7: eluted fractions at 250 mM imidazole. Lane 8: Agarose beads. Proteins were resolved using a Mini-Protean precast TGX 10% gel (Bio-Rad Laboratories) and visualized by Coomassie staining. Loading samples derive from the same experiment and gels were in parallel elaborated. The image is a full-length gel, and it is conform to the original gel.

**Supplementary Fig. 5. Diffraction-data precision indicator (DPI) of the structural models used for comparative analysis**. Crystal structures are identified by their PDB codes and ordered according to their protein type (top) and crystal symmetry (bottom).

**Supplementary Fig. 6. Projection of the matrix of RMSD for 65 chains of COX-1 and COX-2 structures**. Crystal structures are labelled on the top, by using the PDB code, the type (*h*COX-1 or *h*COX-2), the space group (only for *o*COX-1), and the chain letter (A or B, reported only if they do not fall in the same cluster). The chain n.65 (last bin) corresponds to the structure of *h*COX-1 (6Y3C).

**Supplementary Fig. 7. Residue-by-residue root mean square deviation (RMSD) values calculated after superposition of intra-cluster (left) and inter-cluster (right) representative crystal structures**. Intra-cluster representatives are: 5F19-5KIR for *h*COX-2, 5U6X-3N8Z for *o*COX-1 P 6_5_, 2OYE-1U67 for *o*COX-1 P 6_5_22, 1Q4G-2AYL for *o*COX-1 I 222. Inter-cluster representatives are: 5F19-3N8Z for *h*COX-2 *vs* *o*COX-1 P 6_5_, 5U6X-1U67 for *o*COX-1 P 6_5_ *vs* *o*COX-1 P 6_5_22, 3N8Z-1Q4G for *o*COX-1 P 6_5_ *vs* *o*COX-1 I 222, 2AYL-2OYU for *o*COX-1 I 222 *vs* *o*COX-1 P 6_5_22.


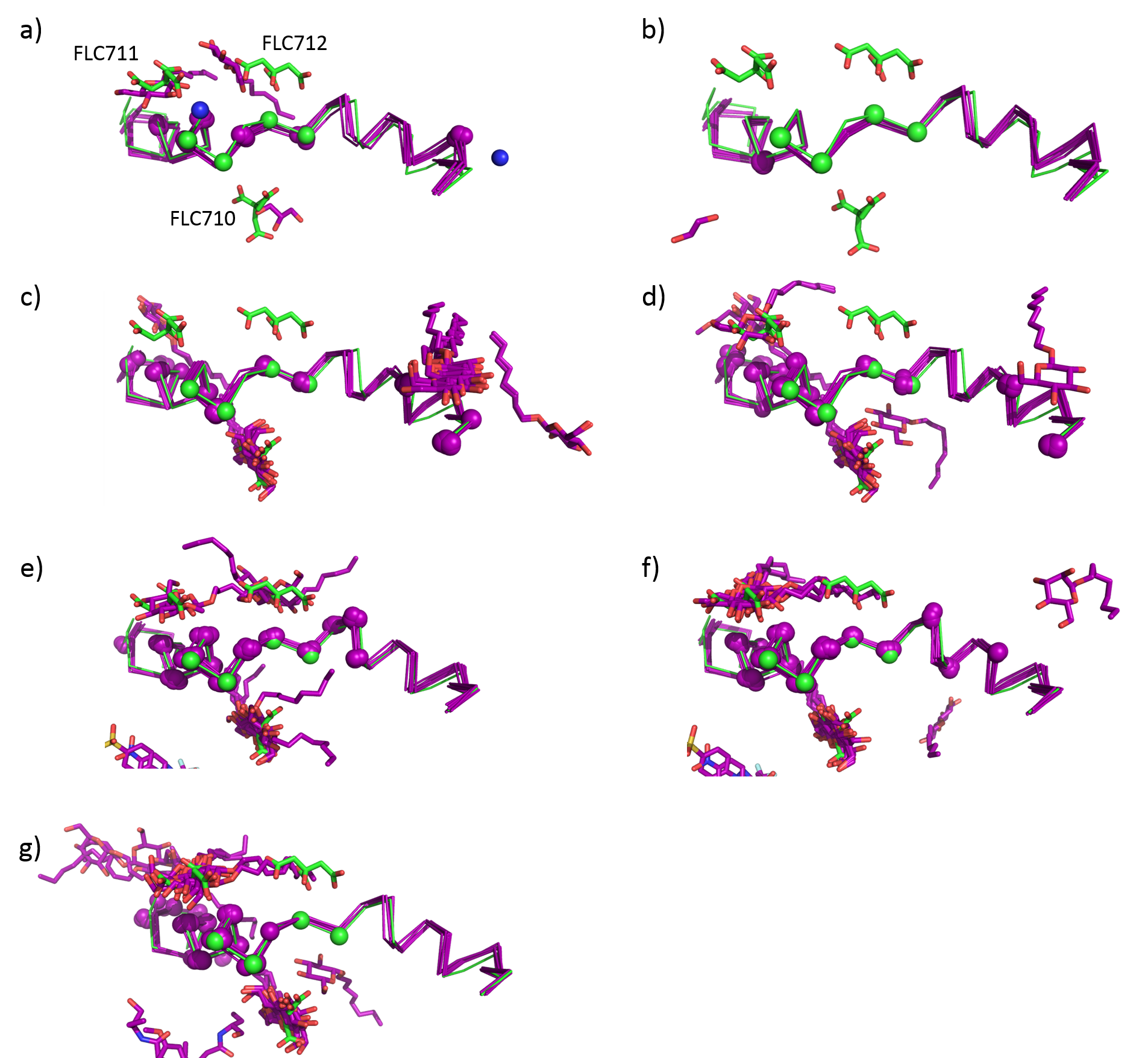


**Supplementary Fig. 8. Interactions between ligands and region 70-110 of COX proteins.** 6Y3C (green) is compared with chains A of *h*COX-2 (a), chains B of *h*COX-2 (b), chains A of *o*COX-1 in I222 (c), chains B of *o*COX-1 in I222 (d), chains A of *o*COX-1 in P6_5_ (e), chains B of *o*COX-1 in P6_5_ (f) and *o*COX-1 in P6_5_ (g). Deposited structures are in purple color. Ammonium ion is shown as blue sphere and, ligand molecules and protein residues are in stick and ribbon representation, respectively. Cα atoms of protein residues closer than 3.5Å to ligand molecules are shown as sphere.

**
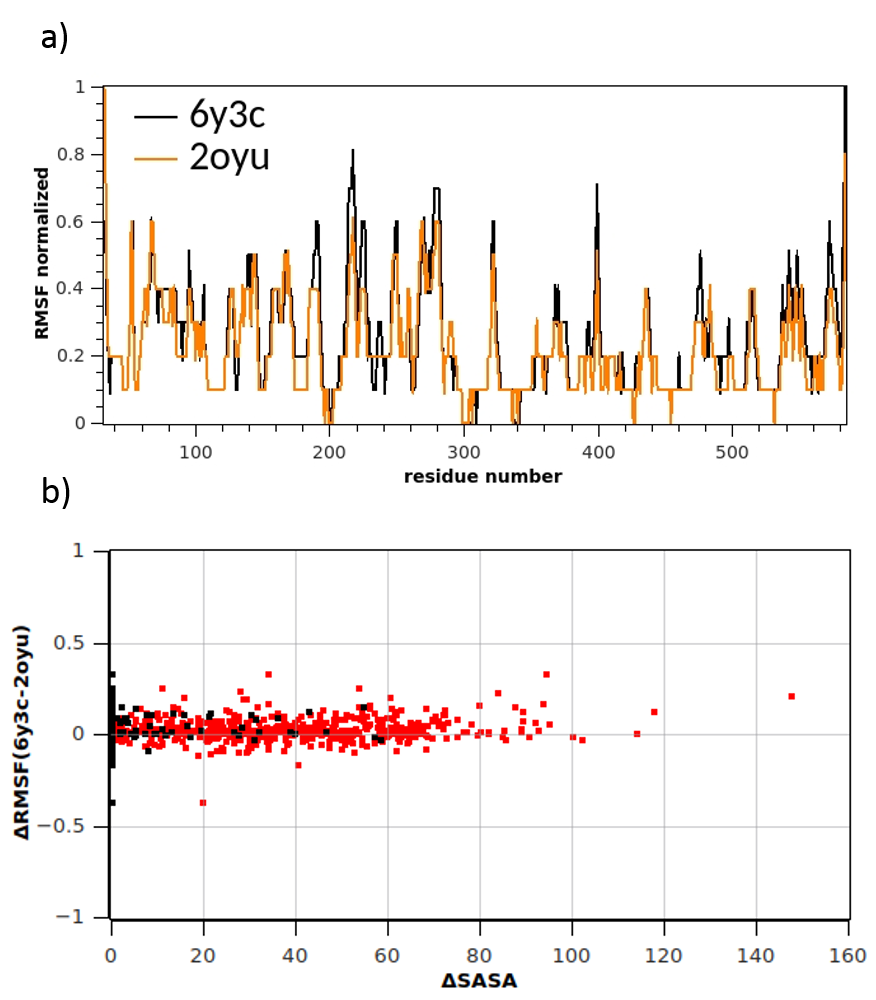
**

**Supplementary Fig. 9 Analysis of protein flexibility.** a) min-max normalized RMSF values for *h*COX-1 (6Y3C) and *o*COX-1 (2OYU) calculated by CABS-flex. b) Differences of RMSF values between 6Y3C and 2OYU plotted against changes of SASA value due to dimer formation (black) or crystal contact formation (red). SASA has been calculated by using only main chain atoms.


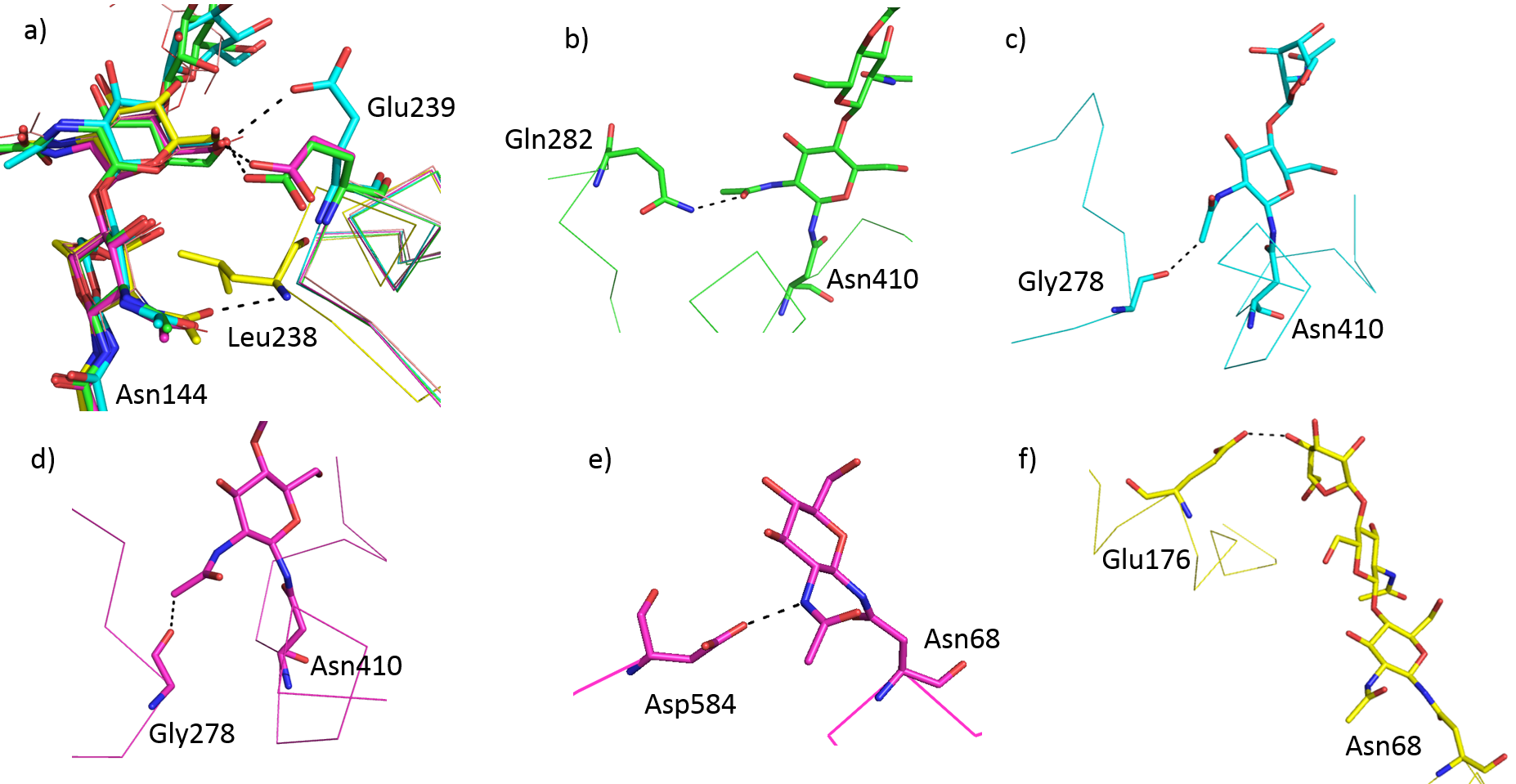


**Supplementary Fig. 10. Inter-molecular interactions involving glycosylation sites.** Hydrogen bonds showing hydrogen donor-acceptor distance less than 3.2Å are shown by dash lines. The crystal structures at highest resolution present in the pdb for *o*COX-1 obtained in P 6_5_22 (2OYU), I222 (1Q4G), and P 6_5_ space group (4O1Z) are shown in cyan, green, and magenta, respectively, and the one for *h*COX-2 in I 222 space group (5F19) is in yellow. *h*COX-1 crystal structure (6Y3C) is in pink. a) Dimer-stabilizing interactions involving glycosylation on Asn144. Residues involved in hydrogen bonds are in stick and the ones not involved in are in line representation. b) Inter-molecular hydrogen bond between Gln282 and glycosylation on Asn410 in the case of *o*COX-1 in I 222 space group (1Q4G). c) Inter-molecular hydrogen bond between Gly278 and glycosylation on Asn410 in the case of *o*COX-1 in P 6_5_22 (2OYU). d) Inter-molecular hydrogen bond between Gly278 and glycosylation on Asn410 in the case of *o*COX-1 in P 6_5_ space group (4O1Z). e) Inter-molecular hydrogen bond between Asp584 and glycosylation on Asn68 in the case of *o*COX-1 in P 6_5_ space group (4O1Z). f) Inter-molecular hydrogen bond between Glu176 and glycosylation on Asn68 in the case of *h*COX-2 (5F19).

**Supplementary Fig. 11. SASA values averaged on conserved residues of the substrate (a) and heme (b) sites**, calculated at increasing probe radii for 6Y3C (*h*COX-1, cyan line), 1U67 (*o*COX-1, green line), and 5F19 (*h*COX-2, black line) crystal structures. Considered residues are Ala199, Ala202, Gln203, His207, Phe210, Lys211, Thr212, Asn382, Tyr385, His386, His388, Val447, Tyr148, and Phe395 for heme site and Val116, Arg120, Phe205, Val228, Leu352, Ser353, Tyr355, Phe381, Leu384, Tyr385, Phe518, Met522, Gly526, Ala527, Leu531, Gly533, Leu534, and Gly526 for substrate site.

**
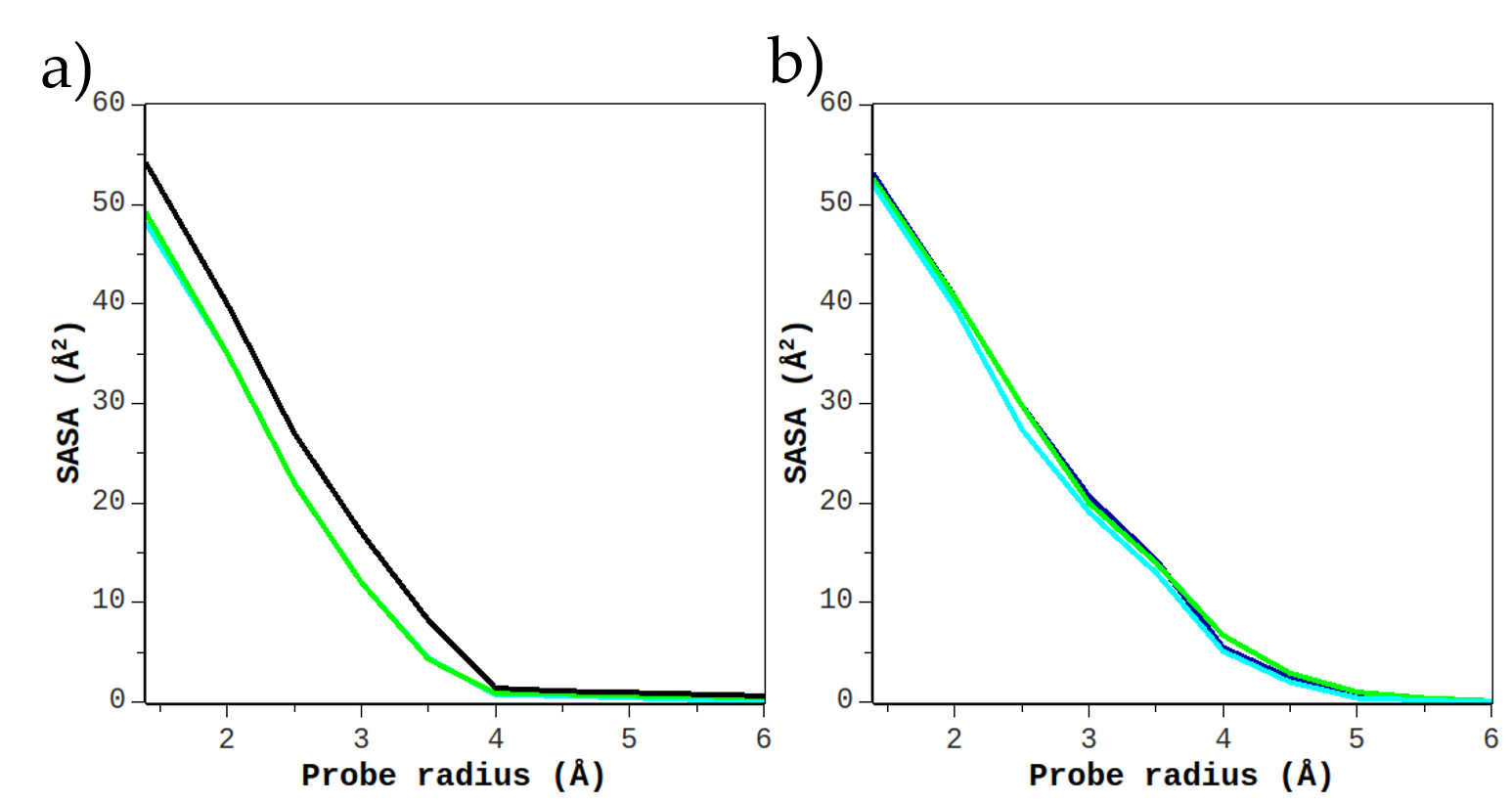
**

**Supplementary Fig. 12. Solvent accessibility of COXs.** *h*COX-1 (green line), *o*COX-1 (cyan line), and *h*COX-2 (black line) after conversion to polyalanine chain. a) SAS of selected residues of the substrate site; b) SAS of selected residues of the heme site.

**Supplementary Table 1**. **Inter-molecular hydrogen bond interactions with glycosylation sites Asn68, Asn144, and Asn410**. Hydrogen bond showing hydrogen donor-acceptor distance less than 3.2Å have been taken into account. The second column shows residues involved in the interaction according to *h*COX-1 sequence. Mutated residues respect to such sequence are marked with an “*”. Residues involved are inter-molecular interactions stabilizing dimer complex, they are marked with “+”.

|  | | 6y3c | 2oyu | 1q4g | | 4o1z | | 5f19 | |
| --- | --- | --- | --- | --- | --- | --- | --- | --- | --- |
|  |  | chain A | chain A | chain A | chain B | chain A | chain B | chain A | chain B |
| Asn144 | LEU238 |  |  |  |  |  |  |  | + |
|  | GLU239 |  | + |  | + |  | + | * | * |
| Asn410 | GLY278 |  |  |  |  |  |  | * | * |
|  | GLN282 |  |  |  |  |  |  | * | * |
| Asn68 | ASP584 |  |  |  |  |  |  | * | * |
|  | LEU176 |  |  |  |  |  |  |  | * |

**Supplementary Table 2**. **Intra-molecular hydrogen bond interactions with glycosylation sites Asn68, Asn144, and Asn410.** Hydrogen bonds showing hydrogen donor-acceptor distance less than 3.2Å have been taken into account. The second column shows residues involved in the interaction according to *h*COX-1 sequence. Mutated residues respect to such sequence are marked with an “*”.

|  | | 6y3c | 2oyu | 1q4g | | 4o1z | | 5f19 | |
| --- | --- | --- | --- | --- | --- | --- | --- | --- | --- |
|  |  | chain A | chain A | chain A | chain B | chain A | chain B | chain A | chain B |
| Asn144 | GLU140 |  |  |  |  |  |  |  |  |
|  | SER143 |  |  |  |  |  |  |  |  |
|  | SER146 |  |  |  |  |  |  |  |  |
|  | TYR147 |  |  |  |  |  |  |  |  |
|  | ARG216 |  |  |  |  |  |  | * | * |
| Asn410 | GLN406 |  |  |  |  |  |  |  |  |
|  | PHE407 |  |  |  |  |  |  |  |  |
|  | LEU408 |  |  |  |  |  |  | * | * |
|  | SER412 |  |  |  |  |  |  |  |  |
|  | MET413 |  |  |  |  |  |  | * | * |
|  | ASP416 |  |  |  |  |  |  | * | * |
|  | GLN400 |  |  |  |  |  |  |  |  |
| Asn68 | GLN42 |  |  |  |  |  |  |  |  |
|  | TYR55 |  |  |  |  |  |  |  |  |
|  | GLY66 |  |  |  |  |  |  |  |  |
|  | PRO67 |  |  |  |  |  |  | * | * |

**Supplementary Table 3**. **Correspondence between hinge points found by comparative analysis based on backbone dihedral angles and protein regions showing positional deviations highlighted by comparative analysis based on Cartesian coordinates**. Hinge points are characterized by the discrimination they introduce among a given ensemble of proteins (information given in Tables 2 and 3), positional deviation regions are characterized by the proteins used for comparison (information given in Fig. 3).

| **Dihedral angles** | | **Positional deviations** | |
| --- | --- | --- | --- |
| **Hinge points** | **Discrimination** | **Residues involved** | **Comparison** |
| 95, 96 | *o*COX-1 P6_5_22, 1PRH among All | 70-100 | *h*COX-1 *vs* *o*COX1 I222 |
| 124, 126 | *h*COX-1 among COX-1 | 125-175 | *h*COX-1 *vs* *h*COX-2 |
| 269, 270, 274, 281, 287 | *o*COX-1 P6_5_22, *h*COX-2, *o*COX-1 I222 among all | 270-290 | *h*COX-1 *vs* all |
| 400, 401, 402 | *h*COX-2 among all | 400-420 | *h*COX-1 *vs* *h*COX-2 |
| 574, 575 | *h*COX-2 among all | 570-584 | *h*COX-1 *vs* *h*COX-2 |

**Supplementary Discussion 1. Interactions between ligands and region 70-110 of COX proteins.**

Both already PDB deposited structures and our *h*COX-1 show ligand molecules closer than 3.5 Å to protein residues included between 70 and 100 (Supplementary Fig. 9). The main difference is represented by the chemical nature of such ligands: surfactant molecules or ammonium ions in the case of deposited structures and citrate ions in the case of our structure. It is noteworthy that the resolution of our structure, low occupancy and flexibility of ligands molecules may have affected map quality and, therefore, it cannot be excluded that other molecules are present instead of citrate ion. The second difference is represented by the localization of such ligands. Particularly, differences appear in the case of *h*COX-2 and *o*COX-1 crystallized in I222. *h*COX-2 structures appear populated by surfactant molecules in the same position of the citrate ion found in our structure only in the case of the chain A of 5IKR structure. Instead, in the case of *o*COX-1 in I222, regardless the chain, only two of the three positions in which citrate ions have been found in our structure show the presence of surfactant molecules. Moreover, *o*COX-1 crystallized in I222 show ligands close to the region 70-73 that is not populated in our case.

**Supplementary Discussion 2. Bioinformatics analysis on COX-1 crystallizability.**

By comparing the primary structure of hCOX-1 and oCOX-1, the presence of a lower content of proline and a higher content of glycine residues in the case of hCOX-1 was observed. Such differences usually result in increased protein flexibility, which in turn reduces the chances to get protein crystals.

To get information on protein flexibility, we used the residue-by-residue Root Mean Squared Fluctuation (RMSF) values calculated by the CABSflex server (http://biocomp.chem.uw.edu.pl/CABSflex2/) both for *h*COX-1 (6Y3C) and *o*COX-1 (2OYU). Moreover, we calculated SASA for *h*COX-1 (backbone atoms only) in the case of protein monomer (SASA_M_), dimer (SASA_D_), and by applying symmetry elements (SASA_C_) for the same proteins. For each residue, the difference of RMSF values between *h*COX-1 and *o*COX-1 (ΔRMSF_6Y3C-2OYU_) has been plotted against ΔSASA_MD_ = SASA_M_-SASA_D_ and ΔSASA_MC_ = SASA_M_-SASA_C_ (Supplementary Fig. 8). We observed that residues showing the largest value of |ΔRMSD6Y3C-2OYU| also show large ΔSASAMC values. Such result points out that the residues characterized by the largest difference in flexibility between the two proteins are involved in crystal contacts. Instead, such residues are not involved in dimer formation, as they have negligible ΔSASAMD values. These results could explain why the two proteins show similar activity (dimer formation is not affected by differences in protein flexibility) and different crystallizability.

Although small, differences of hydrophobicity and surface entropy have been detected for these two proteins. The “Detect hydrophobic patches” tool of Swiss PDB shows that the area of the first 13 largest hydrophobic patches corresponds to 11 % and 10 % of the total protein surface for hCOX-1 and oCOX-1, respectively. This suggests a slightly higher tendency of hCOX-1 to aggregate instead to remain in solution, a characteristic that increases the probability of non-specific aggregation and reduces the chance of crystallization. The SER server (http://services.mbi.ucla.edu/SER/) shows a higher surface entropy for hCOX-1 than oCOX-1 for the regions 326-336 and 455-460, the former of which being part of the dimer interface. No differences between the two proteins have been detected by bioinformatics analysis based on primary structure, such as the calculation of the probability of coiled regions by the COILS2 server (https://embnet.vital-it.ch/software/COILS_form.html) or the index of crystallizability by the XtalPred server (http://xtalpred.godziklab.org/XtalPred-cgi/xtal.pl). Therefore, even if the differences we found are not enough to draw firm conclusions, the higher disorder/flexibility of hCOX-1 with respect to oCOX-1 could explain why the former protein is more recalcitrant to crystallize.
